# Supramolecular Self-Assembled Chaos: Polyphenolic Lignin’s Barrier to Cost-Effective Lignocellulosic Biofuels

**DOI:** 10.3390/molecules15128641

**Published:** 2010-11-29

**Authors:** Komandoor Elayavalli Achyuthan, Ann Mary Achyuthan, Paul David Adams, Shawn Matthew Dirk, Jason Carl Harper, Blake Alexander Simmons, Anup Kumar Singh

**Affiliations:** 1 Joint BioEnergy Institute (JBEI), Emeryville, CA 94550, USA; 2 Sandia National Laboratories, Albuquerque, NM 87185, USA; E-Mails: smdirk@sandia.gov (S.M.D.); jcharpe@sandia.gov (J.C.H.); 3 Biology Department, Northern New Mexico College, Espanola, NM 87532, USA; E-Mail: annachyuthan@nnmc.edu (A.M.A.); 4 Lawrence Berkeley National Laboratory, Berkeley, CA 94720, USA; E-Mail: pdadams@lbl.gov; 5 Sandia National Laboratories, Livermore, CA 94550, USA; E-Mails: basimmo@sandia.gov (B.A.S.); aksingh@sandia.gov (A.K.S.)

**Keywords:** lignin, supramolecular self-assembly, cell wall, deconstruction, enzymes, LCC, fractal, lignocellulosic biofuels

## Abstract

Phenylpropanoid metabolism yields a mixture of monolignols that undergo chaotic, non-enzymatic reactions such as free radical polymerization and spontaneous self-assembly in order to form the polyphenolic lignin which is a barrier to cost-effective lignocellulosic biofuels. Post-synthesis lignin integration into the plant cell wall is unclear, including how the hydrophobic lignin incorporates into the wall in an initially hydrophilic milieu. Self-assembly, self-organization and aggregation give rise to a complex, 3*D* network of lignin that displays randomly branched topology and fractal properties. Attempts at isolating lignin, analogous to archaeology, are instantly destructive and non-representative of *in planta*. Lack of plant ligninases or enzymes that hydrolyze specific bonds in lignin-carbohydrate complexes (LCCs) also frustrate a better grasp of lignin. Supramolecular self-assembly, nano-mechanical properties of lignin-lignin, lignin-polysaccharide interactions and association-dissociation kinetics affect biomass deconstruction and thereby cost-effective biofuels production.

## 1. Introduction

**Figure 1:**
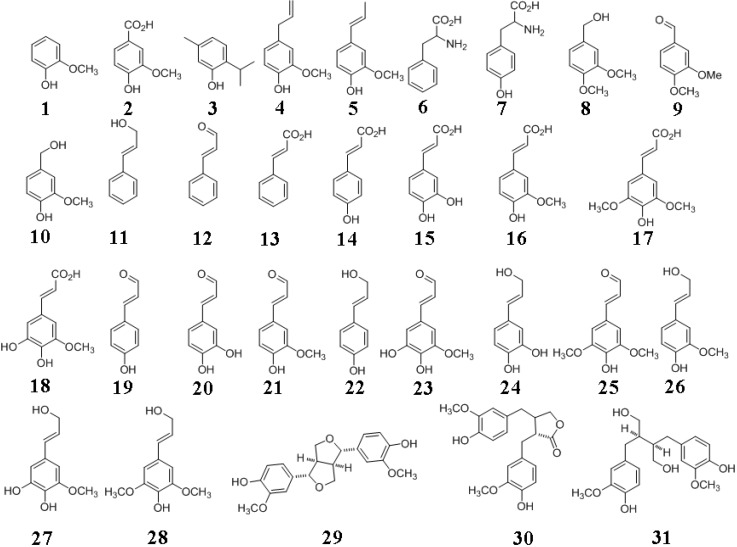
Structures of phenols relevant to lignin. **1**, guaiacol; **2**, vanillin; **3**, thymol; **4**, eugenol; **5**, isoeugenol; **6**, phenylalanine; **7**, tyrosine; **8**, veratryl alcohol; **9**, veratraldehyde; **10**, vanillyl alcohol; **11**, cinnamyl alcohol; **12**, cinnamaldehyde; **13**, cinnamic acid; **14**, *p*-coumaric acid; **15**, caffeic acid; **16**, ferulic acid; **17**, sinapic acid; **18**, 5-hydroxyferulic acid; **19**, *p*-coumaraldehyde; **20**, caffeoyl aldehyde; **21**, coniferaldehyde; **22**, *p*-coumaryl alcohol; **23**, 5-hydroxyconiferaldehye; **24**, caffeoyl alcohol; **25**, sinapaldehyde; **26**, coniferyl alcohol; **27**, 5-hydroxyconiferyl alcohol; **28**, sinapyl alcohol; **29**, (+)-pinoresinol; **30**, matairesinol; **31**, (-)-secoisolariciresinol. Phenols **29** to **31** are lignans.

Plants synthesize several thousand different types of phenolic compounds, including simple phenols and polyphenols [1]. Lignin is a phenolic polymer that constitutes a major barrier against cost-effective lignocellulosic biofuels by complexing with cellulose and preventing hydrolytic enzymes (cellulase, β-glucosidase) from accessing the sugar and by non-productively adsorbing such enzymes on the hydrophobic lignin surface. The structures of some phenols relevant to lignin are shown in Figure 1. 

In order to contextualize the barrier properties of lignin, we review lignin formation beginning with the biosynthesis of its phenylpropanoid subunits and subsequent non-enzymatic polymerization of the phenyl units (monolignols; Figure 1), and ending with the incorporation of lignin into the plant wall. The phenylpropanoids are so called because of their phenyl ring (C6) and propane (C3) side chain. The numbering of monolignols begins with the side chain carbon being C1 and the aromatic carbon bound to the -OH group being C4. The -OCH_3_ group has a low number; *i.e.*, the carbon atom would be C3 with a single -OCH_3_ substituent, and not C5. The side chain carbons are designated as α, β and γ.

In addition to lignin-carbohydrate complexes, we also summarize the less prominent lignin-protein interactions and briefly describe lignans that are typically made up of two phenylpropane units (Figure 1). We list methods for lignin analyses and describe imaging technologies for the plant cell wall. We discuss key microbial enzymes involved in lignin degradation. Lignin barrier properties are remarkable from the fact that although about 100 billion tons of biomass is produced yearly, equaling about five-fold the annual worldwide energy consumption, less than 1% of this energy source is utilized commercially [2]. While there is considerable optimism about biofuels, truly cost-competitive lignocellulosic biofuels are years away. We hope that this review will serve as an introduction to lignin, its role in cell wall assembly and assist in developing environmentally-friendly biomass deconstruction strategies for producing cost-effective lignocellulosic biofuels. We strived to balance current citations, original papers and review articles, while remaining concise. We regret the inevitable bias in the reference list, compelling us to choose from among thousands of publications in the field. 

## 2. Plants

Since this review is about plant phenolics, it is appropriate to provide an overview of plants. The plant characteristics described below also affect biofuel strategies, a central theme of this review. Land plants evolved about half a billion years ago, presumably from green algae. Green algae are aquatic organisms that utilize nutrients present in the water along with sunlight to photosynthesize life’s essentials. Unlike land plants, green algae do not possess a vascular system for the transport of nutrients and gasses. Bryophytes such as moss or liverwort also do not possess a vasculature even though they are land plants. Non-vascular plants lack roots, stems and leaves. Vascular plants were originally exemplified by ferns and horsetails that culminated in seed-bearing land plants such as conifers about 300 million years ago [3]. Plant vasculature is relevant to this review since its water conducting xylem is the site for lignin deposition. Flowering plants followed the seed-bearing plants. The flower is a plant’s reproductive element with seeds containing the ovary which develops into a fruit. The presence or absence of ovary results in the plants being classified as angiosperms (flowering plants) or gymnosperms (“naked” seeds). The wood from these two types of plants are called hardwood and softwood, respectively. Wood is the secondary xylem thickening due to lignin deposition which is familiar as the “tree rings” that are used to date a plant’s lifetime. Wood evolved after plants attained a terrestrial distribution and woody plants became seed-bearing plants. By contrast, non-woody plants contain only a primary xylem. All “true” woody plants are perennials, living for >2 years, in contrast to annuals and biennials; not all perennials are woody though. For example, a coconut palm lacks the secondary xylem and is therefore not considered a woody plant. Some properties of gymnosperms and angiosperms are summarized in Table 1. There is yet another class of plants, the *Gramineae* (grasses; *Poaceae*). Grass lignin is discussed under various sections of this review.

**Table 1:** Phenotypic properties of Gymnosperms and Angiosperms.

Gymnosperms	Angiosperms
*Softwoods*	*Hardwoods*
Non-flowering (some exceptions)	Flowering monocotyledons (*eg.,* corn) and dicotyledons (eg., beans)
Non-fruiting trees	Fruit trees
Coniferous “ever greens” (some conifers are deciduous) – conifers are major gymnosperms	All woody angiosperms are dicotyledons (not all dicotyledons are woody)
“Naked” seeds; bear cones	Seeds covered in fruit or nut
Retain/shed leaves throughout the year	Shed leaves at one particular time of year
Needle shaped leaves mostly	Well formed leaf structure
Cedar, Fir, Pine, Spruce, Redwood, Juniper, Cypress, Giant Sequoia, *etc.*	Ash, Mahogany, Oak, Aspen, Walnut, Balsa, Elm, Birch, Maple, *etc.*
Temperate growth regions	Temperate/tropical growth regions
Lower density wood	Higher density wood
Less expensive	More expensive
Smaller group (~20% of plant kingdom)	Largest group (~80% of plant kingdom)
Evolutionarily “primitive”	Evolutionarily “advanced”
Evolutionarily first seed-bearing plants	Seed plants evolved later
Oldest and largest trees (eg., giant sequoias)	More recent and smaller trees mostly
Reaction wood is mostly compression wood	Reaction wood is mostly tension wood

## 3. General Properties of Lignin

Lignin is the second most abundant biopolymer on Earth, comprising 15 to 25% of biomass and exceeded only by polysaccharides, of which heteropolysaccharides (hemicellulose) account for 20 to 30% with the balance (35 to 50%) being made up of cellulose. Small amounts (1 to 5%) of ash, extractives, and nitrogenous materials have been reported as plant cell wall constituents [4]. Lignin is a fascinating molecule where established paradigms are constantly being overturned by new discoveries. Lignin’s complexity, degree of polymerization and diversity are so great that there may be no two identical lignin macromolecules with the same primary sequence of phenyl units. Therefore, it is more appropriate to refer to these biopolymers in the plural as “lignins.” Furthermore, a useful way to define lignin is to list its properties due to the difficulties in finding a generally applicable/acceptable definition [5,6]. It was suggested that the diversity of lignins was so great as to render the sequencing of lignin molecules of limited value [7]. Yet, the primary sequence of a lignin molecule was published recently [8], illustrating the constantly changing facets of our understanding of lignin’s biology. 

Lignin is derived from the Latin term *lignum*, meaning wood. Lignin is a precursor to the fossil fuel, coal. Lignin is frequently described as a random, complex, irregular, heterogenous, 3*D*, varyingly branched network of crosslinked, phenolic (aromatic) biopolymer. Nevertheless, the degree of crosslinking may not be high depending on the wood source [4,9]. Lignin is composed of three main phenylpropane units (hydroxycinnamyl alcohols that vary in their degree of methoxylation: coniferyl, sinapyl, and *p*-coumaryl alcohols) (Figure 1), in addition to several different minor phenolic compounds. Even the molecular mass of lignin is debated, ranging from tens of thousands of Daltons to essentially the infinite [10]. Lignins are racemic and therefore optically inactive. The racemic nature of lignins might arise from the fact that its polymerization is a non-enzymatic process [11]. Lignins are amorphous, hydrophobic heteropolymers and display variations in both chemical composition and structure due to a low degree of order and a high level of heterogeneity. These properties make the isolation of unaltered lignin for structural and/or compositional analyses a challenging task. It has not yet been possible to isolate the entire lignin fraction from plant cell walls in a pure form since the various physico-chemical techniques used for breaking down the walls also cause substantial alterations to lignin structure [12]. In this sense, a study of lignins, akin to archaeology, is instantly destructive. There is a concern that the isolated lignins might not represent the cell wall *in planta*. At this writing, ball milled wood lignin (MWL), isolated from finely powdered wood by the action of mild, neutral solvents, is considered to be the closest to *in vivo* lignin [13,14]; even this lignin’s recovery is low and the isolated lignin has different degrees of polysaccharide contamination and may be depolymerized to varying extents due to the reactivity of the benzylic ether bond and also cleavage of aryl-ether bonds [15]. The isolated lignin is called “technical lignin” in order to distinguish it from *in vivo* lignin. For these reasons, and in contrast to the polysaccharides, lignin is an under-appreciated bioresource. However, this situation is changing with the isolation of pharmacologically useful derivatives of lignin, with the result that it is gaining importance and progressively less lignin is being burnt simply for fuel [2]. 

Lignins are natural glues that bind tightly to polysaccharides, complicating our understanding of its native structure. Due to its hydrophobic character, lignin makes plant cells impermeable to water. Lignins also provide mechanical rigidity enabling plants to defy gravitional forces and grow skyward, affording their sometimes immense size and volume (such as giant redwoods, sequoias). Lignins enable vascular integrity and protect the cell wall polysaccharides, which makes it difficult to exploit the sugars for biofuel. Lignins offer protection against pathogens, pests and natural or mechanical wounding. However, the chemical composition of such stress-induced lignin is different from the lignin that is deposited during the normal formation of a cell wall by being higher in *p*-coumaryl alcohol content [16]. The vital properties of lignin are evidenced from the impaired growth and viability of plants with low or disorganized lignin (naturally or due to genetic engineering) [17,18]. Lignin enables the transport of water from the roots to the leaves through the xylem vasculature. The lignified cell walls are essentially dead cells linked together to form long hollow tubes. The inside of these tubes are the sites for lignin deposition (*i.e.*, lignification) [6]. Lignin is deposited in the secondary walls and among the fibers amidst non-lignified tissues. Such polydisperse deposition also hinders the isolation and characterization of pure, intact lignin.

Lignin accounts for about one-third of the organic carbon on Earth, or approximately, 10^12^ kilograms [18]. Lignin synthesis starts with the energetically-costly biosynthesis of the three major monolignols (Figure 1). Despite this expense [19], plants have not yet been reported to possess delignifying enzymes that are capable of depolymerizing or degrading lignin [6,20,21,22]. In addition to a number of distinguishing features of lignin, an absence of authentic plant-derived “ligninases” is unique since bio-macromolecules such as nucleic acids, proteins, polysaccharides and lipids are routinely recycled by endogenous cellular enzymes. The chemical bonds in lignin are sufficiently varied that they are perhaps incapable of being recognized and degraded by a single enzyme [23]. These factors make lignin not only a one-way carbon sink but also raise the issue of how plants achieve metabolic balance [19,24,25]. Lignins were originally thought to have evolved with pteridophytes (example, ferns), amongst the earliest vascular land plants some 450 million years ago. Even this notion was overturned by the discovery of lignin in the red algae *Calliarthron cheilosporiodes* [26]. 

Despite intense investigations over 100 years, the sequence of phenolic units and the structure of lignin remain largely a mystery. The complexity of lignin macromolecule and its tight association with polysaccharides have made lignin purification exceedingly difficult and its intact isolation from the plant cell wall nearly impossible and instantly destructive. The levels of lignin and its chemistry vary significantly between various species of plants, among individual plants of the same species, among various parts within a single plant (such as a leaf or stem), and even between various tissues and cells of a single plant (Table 2) [5,12,16,23,27,28]. Lignin content also varies depending on the developmental stage of a plant [28]. For example, lignin composition reaches higher syringyl/guaiacyl (S/G) ratios as plants attain maturity [29]. Lignin composition also changes in response to external and natural, environmental or artificial stressors including, drought, low temperature, ultraviolet irradiation, mineral deficiency, mechanical wounding, and attack by pathogens and pests [30]. For example, plants have been shown to produce lignin with enhanced levels of *p*-coumaryl alcohol at the sites of injury. Several properties of lignin affecting biomass deconstruction are listed in Table 2.

**Table 2:** Comparative properties of softwood and hardwood lignins.^ *^

Softwood Lignin	Hardwood Lignin
Lignin content is ~ 28%	Lignin content is ~ 20%
Lignin dissociates faster in solution	Lignin dissociates slower in solution
Lignin self-associates greater in solution	Lignin self-associates less in solution
Harder to breakdown lignocellulosic biomass	Easier to breakdown lignocellulosic biomass
Coniferyl alchol primarily (~80%)	Coniferyl (~56%) and Sinapyl (~40%) alcohols
Guaiacyl (coniferyl alcohol derived) G-lignin	Guaiacyl-Syringyl (G-S) lignin; Syringyl is sinapyl alcohol derived lignin
Gymnosperms	Angiosperms, Dicotyledons
Molecular mass is larger than hardwood lignin	Molecular mass is lower than softwood lignin
Branching is higher	Branching is lower; Lignin is more linear
Cross-links are greater	Cross-links are fewer
C-C bonds are greater	C-C bonds are fewer
5' Linkages more common	5' Linkages less common
–OCH_3_ content is ~20%	–OCH_3_ content is ~14%
β-O-4 ether bonds are lower	β-O-4 ether bonds are higher
β-β and β-5 bonds are higher	β-β and β-5 bonds are fewer
Deconstruction is harder	Deconstruction is easier
Lignin is condensed	- - - - -

^*^ *p*-Coumaryl alcohol (*p*-hydroxyphenyl) derived lignin (H-Lignin) is more common among *Graminaceous* plants (grasses). The –OCH_3_ content is nearly zero in H-lignin. Monocotyledon angiosperms contain G-S-H lignin. Guaiacyl unit has one methoxy group whereas syringyl unit has two methoxy groups.

## 4. Lignin Formation

Lignin formation is a complex process since in addition to engaging physiological and biochemical processes there are also non-biological, spontaneous chemical activities controlling its formation. Lignin synthesis is preceded *primarily but not exclusively*, by the biosynthesis of its basic units, the three monolignols (Figure 1). Lignin formation involves: a) biosynthesis of monolignols; b) transport of monolignols to lignifying sites; c) enzymatic radicalization of monolignols; and, d) non-enzymatic coupling of the radical monomers into the growing lignin polymer [31]. This is a simplified description of lignin formation; several aspects of even these simple steps are controversial. 

### 4.1. Monolignol Biosynthesis

**Figure 2:**
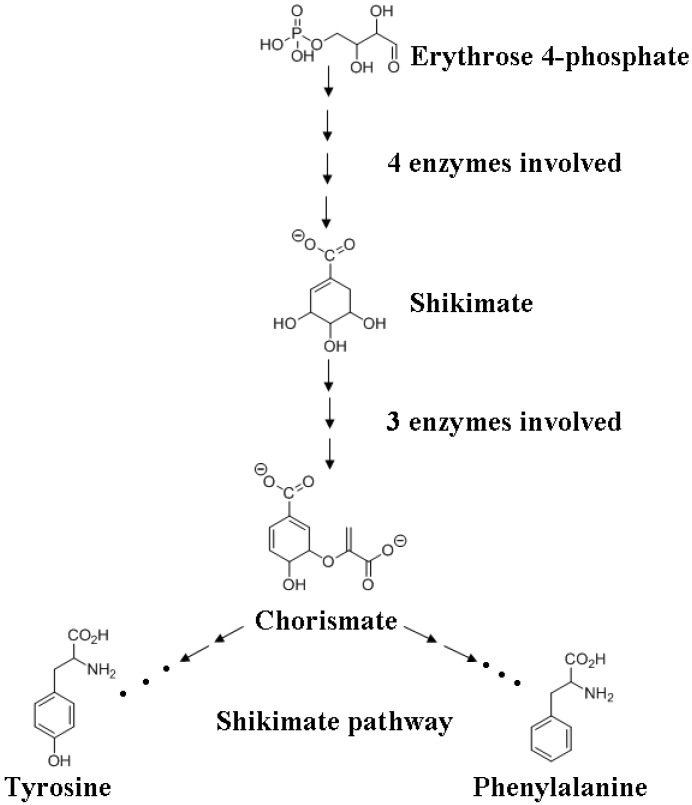
Overview of the shikimate pathway for the biosynthesis of phenylalanine and tyrosine.

*p*-Coumaric acid undergoes a series of reactions termed the phenylpropanoid pathway in order to generate the three monolignols with varying methoxy substitutions (Figure 1) [32]. The genealogy of lignin extends from fully formed lignin associated with the heteropolysaccarides of a plant cell wall back to lignin’s origins from the monolignols, which in turn is preceded by the shikimate pathway starting with yet another carbohydrate, erythrose 4-phosphate [6,12] (Figure 2). 

It is from carbohydrates that the amino acids phenylalanine and tyrosine (Figure 1) are generated that in turn transform into the monolignols catalyzed by a series of enzymatic steps. A detailed discussion of the shikimate pathway is beyond the scope of this review but reference 33 may be consulted for more information. Briefly, one of the products of carbohydrate metabolism, erythrose 4-phosphate, is converted to shikimate in four distinct enzymatic steps. Shikimate is transformed into chorismate by the actions of three different enzymes. Chorismate undergoes a series of reactions to yield tyrosine or phenylalanine, the precursors of hydroxycinnamyl alcohols (monolignols) (Figure 2). 

**Figure 3:**
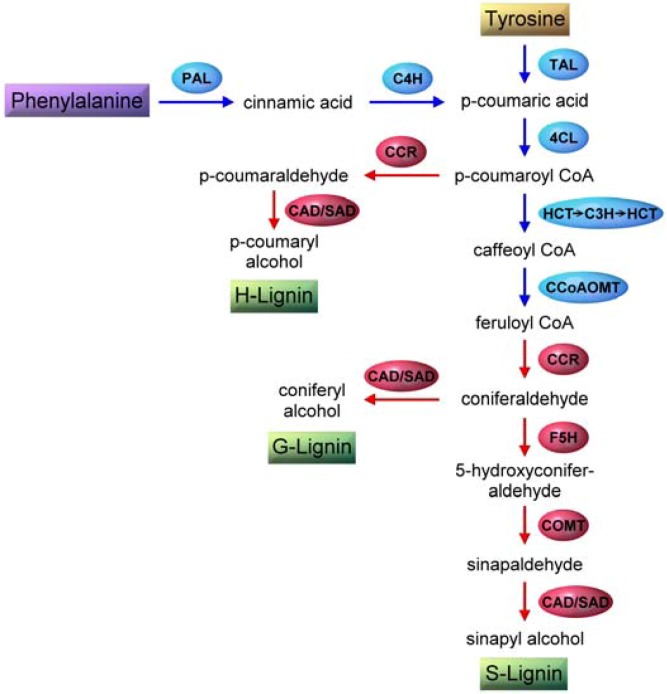
Outline of enzymes/pathways involved in monolignol biosynthesis. Enzymes are enclosed in ellipses while substrates and products are boxed. Phenylpropanoid pathway is shown in blue and monolignols biosynthesis in red. Arrows indicate sequential enzymatic steps. Abbreviations are (order of appearance): PAL, phenylalanine ammonia lyase; C4H, cinnamate 4-hydroxylase; TAL, tyrosine ammonia lyase; 4CL, 4-coumarate-CoA ligase; CCR, hydroxycinnamoyl-CoA reductase; CAD, coniferyl alcohol dehydrogenase; SAD, sinapyl alcohol dehydrogenase; HCT, *p*-hydroxycinnamoyl-CoA:quinate shikimate p-hydroxycinnamoyl-CoA transferase; C3H, *p*-coumarate 3-hydroxylase; CCoAOMT, caffeoyl-CoA *O*-methyltransferase; F5H, ferulate 5-hydroxylase (coniferaldehyde 5-hydroxylase); COMT, caffeic acid/5-hydroxyferulic acid *O*-methyltransferase.

A simplified view of the biosynthesis of the three major monolignols is shown in Figure 3. It begins with the enzymatic deamination of phenylalanine by phenylalanine ammonia lyase (PAL) [34,35,36]. This is followed by a series of reactions catalyzed by hydroxylases (hydroxylation), methyl transferases (methylation) and dehydrogenases (reduction) [28]. Several plants including grasses express tyrosine ammonia lyase (TAL) activity which skips a step in the phenylpropanoid pathway [32] (Figure 3). It is likely that certain PAL enzymes have broad specificity and accept tyrosine as substrate, combining PAL and TAL activities within a single enzyme. It is also possible that there are dedicated TAL enzymes in plants. The enzymes that are amenable to molecular biological modifications in order to deposit lignin which is easier to break down (Table 2) are also shown in Figure 3. There are gaps in our understanding of monolignol biosynthesis such as the presence of additional, unidentified enzymes or the invoking of a metabolic grid of intersecting/alternative pathways or independent channels for their biosynthesis and also regarding the exact role of TAL [12,17,29,30,36].

### 4.2. Monolignol Transport

The next step in lignin formation is the transport of monolignols to lignification sites during xylogenesis (wall development). Despite years of study, monolignol transport is unclear [9,16,37]. However, it is a key step in lignin formation with significant implications for biomass deconstruction and cost-effective lignocellulosic biofuels production. Lignification can be modified or disrupted by interfering with the transport processes. Ralph *et al.* [5] emphasized the need for more research on monolignol transport mechanisms, since lignin formation is controlled by the supply of the monomers to the lignifying zones. Phenylpropanoid glycosides [38] such as coniferin and syringin (glycosides of coniferyl and syringyl alcohols) have been synthesized [39] and also been isolated in high amounts from plant tissues [38], leading to suggestions that these compounds might be the storage and/or transport forms of the corresponding monolignols [12]. Glucosyl transferases [38] and β-glucosidases might regulate the availability of monolignols during lignification [40,41]. Cell wall-associated β-glucosidases might release the aglycone (monolignols) for subsequent polymerization during lignin synthesis. An alternate pathway was proposed involving coniferaldehyde glucoside that was converted to coniferyl alcohol or coniferaldehyde before becoming incorporated into lignin [42].

These views were challenged from biosynthetic studies of radiolabeled phenylalanine administered to lodgepole pine during plant development. Based on the tracer’s fate in tissues, Kaneda *et al.* [43] suggested that “unknown membrane transporters, rather than Golgi vesicles, export monolignols.” One reason for the uncertainty regarding the direct transport of monolignols is that these compounds are hydrophobic and must travel through the hydrophilic cellular matrix in order to reach the sites of lignin deposition. Another reason for the difficulty lies in the nature of lignin itself with several different types of phenolic units (discussed below) besides the three major monolignols becoming incorporated into the biopolymer [22]. For this reason, it was suggested that not only “free” (native, unmodified) coniferyl and sinapyl alcohols diffuse through the plasma membrane, but that non-specific transporters are also involved in conducting the monolignols to the cell wall rather than glycosyl transferase- and β-glucosidase-mediated transport and release of phenylpropanoid glycosides [7,27,35]. All this leaves the role of phenylpropanoid glycosides in plant physiology opaque. It is particularly important since lignin is a metabolically expensive, one-way carbon sink [19,24] and therefore the plants must carefully control its synthesis [25]. If phenylpropanoid glycosides do not participate in monolignol transport to lignification sites, then why do plants synthesize these molecules? The antimicrobial and antioxidant activities of phenylpropanoid glycosides are partial answers to this question [44]. This may not be the entire explanation for compounds such as coniferin and syringin [35]. As emphasized above [5], monolignol transport requires further research.

### 4.3. Monolignol Radical Formation

**Figure 4:**
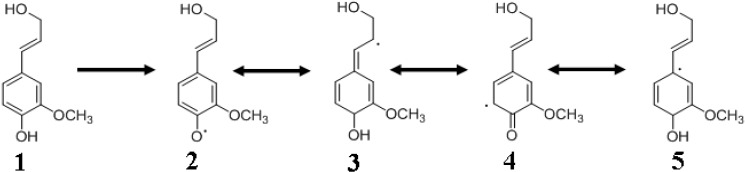
Coniferyl alcohol and its radicals. **1**, coniferyl alcohol; **2** and **4** are radicals; **3** and **5** are quinone methide radicals. First step is coniferyl alcohol radicalization by enzymatic dehydrogenation.

Once the monolignols are transported to lignification sites, they undergo radicalization. Redox enzymes such as peroxidases, phenol oxidases and laccases have been implicated in the oxidative radicalization of the monlignols [23,27,28]. Redox shuttle mediators (RSM) [45] sometimes involving the monolignols (coniferyl or veratryl alcohols) themselves, were also implicated through exchange reactions [28,46,47], in the radicalization of monolignols or for the polymerization of lignin [28]. The resonance stabilized radical structures of coniferyl alcohol are shown in Figure 4. The precise classes of all the enzymes or the role of specific isoenzymes involved in the monolignol radicalization have not been elucidated. This is partly due to the broad substrate specificity of these redox enzymes, making it difficult to pinpoint enzymes catalyzing monolignol radical formation [5,48,49]. For example, almost all peroxidases can oxidize coniferyl alcohol efficiently and also oxidize sinapyl alcohol, albeit at lower rates [23]. However, plants compensate for this disadvantage by expressing sinapyl alcohol-specific peroxidases [50]. 

The formation of resonance-stabilized radicals of coniferyl alcohol begins with its enzymatic dehydrogenation and the formation of a free radical at the 4-hydroxyl position (Figure 4). Other resonance structures are also formed, leading to the generation of quinone methide intermediates. These radicals are quite stable due to electron delocalization, resulting in single electron density at the phenolic ring as well as on the side chain β-carbon (Figure 4) [37]. The substituents on the monolignol determine the decay of its radical cation. Electron-rich groups favor the formation and stabilization of the radical cation [51]. Despite considerable uncertainty regarding monolignol transport and polymerization, the enzymatic radical generation has not been questioned. Laccases utilize oxygen for oxidation whereas peroxidases utilize hydrogen peroxide (H_2_O_2_) for oxidizing the monolignols. A number of different oxidases were proposed for H_2_O_2_ generation at the lignification sites in order to assist in the peroxidase-catalyzed monolignol radicalization [28]. Cell wall-associated peroxidases and laccases might additionally enable lignin polymerization at specific sites.

### 4.4. Monolignol Polymerization

Perhaps no aspect of lignin formation has been characterized by more controversy than the polymerization of monolignols to yield the lignin macromolecule. It has even been described as a “war” [52]. A series of papers by Lewis and colleagues have proposed that dirigent (from the Latin word *dirigere*, which means to ‘guide’ or to ‘align’) proteins are involved in lignin polymerization and determining its stereochemistry [9]. According to these authors, the dirigent proteins are nonenzymatic and do not participate in radical generation but instead control the polymerization and the coupling of the monolignol radicals into the growing lignin polymer using a template mechanism resulting in one lignin chain enabling the synthesis of its own mirror-image chain [53]. Using a computational approach, a double stranded lignin template was proposed to enable the placement of monolignol radicals upon the template, resulting in the synthesis of a new lignin chain whose phenolic units sequence was identical to the template strand [54].

These ideas have been refuted by the random model theory, championed by Ralph and others, arguing that the coupling of monolignol radicals is governed by chemical and environmental factors at the lignifying sites, and that coupling is not under any kind of protein control [7,27]. Such factors included the type, concentration and the rate of monolignols arriving at lignification sites, the reaction propensities of the monolignols, availability of redox enzymes and H_2_O_2_, matrix in which lignification was taking place, and the reaction milieu of pH, ionic strength, temperature, *etc.* The term “combinatorial” was used to describe the random nature of the coupling reactions [5,28] resulting in lignin polymers that had no fixed or unique sequence of the phenylpropanoid units. This type of polymerization explained the extensive structural diversity of natural lignins and its racemic nature [11,55]. The random, chemically-driven polymerization process also meant that besides the three major monolignols, any other phenolic radical in the vicinity of the growing polymer could become incorporated into lignin [5]. It has also been asserted that no natural lignin is composed of solely the three monolignols [22]. Indeed, *p*-coumarate (grass lignin) [56], hydroxycinnamic acid amides [57], *p*-hydroxybenzoate [58,59], ferulic acid, 5-hydroxyconiferyl alcohol, hydroxycinnamate esters, hydroxycinnamaldehydes, hydroxybenzaldehydes, coniferaldehyde, sinapaldehyde, dihydroconiferyl alcohol (Figure 1) and other types of phenolics were found to be crosslinked to lignin conferring considerable flexibility and plasticity to lignin synthesis, its structure and primary sequence [5,12,22,23,35].

**Figure 5:**
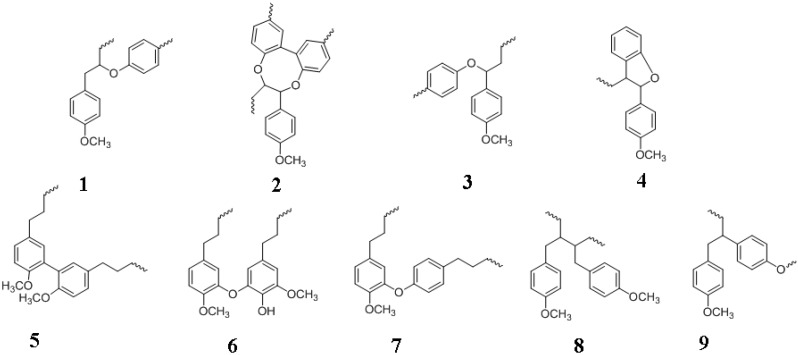
Lignin crosslinks. **1**, 8-O-4/β-O-4 phenylpropane β-arylether; arylglycerol-β-arylether; ~50% softwood lignin; **2**, dibenzodioxocin; ~20% softwood lignin along with 5-5 links; **3**, phenylpropane α-arylether; ~7% softwood lignin; **4**, phenylcoumaran; ~10% softwood lignin; **5**, 5-5, biphenyl; ~20% along with dibenzodioxocin; **6**, 5-O-4 biphenylether; ~6% softwood lignin; **7**, 4-O-5 diarylether; **8**, β-β, pinoresinol; ~3% softwood lignin; **9**, 1,2-diarylpropane-1,3-diol; ~8% softwood lignin.

The most abundant phenylpropane linkage in softwood and hardwood lignins is β-O-4 (50 to 80%). Other linkages of lignin include α-O-4, β-5, β-β, β-1, 4-O-5, 5-5 and dibenzodioxocin [60] with the last three serving as branching points [15]. More than twenty different linkage types have been identified and it is thought that several more will be discovered as lignin chemical analyses progresses [22]. Branching requires the crosslinking between two preformed lignins at their phenolic ends. Guaiacyl unit is also mandatory for crosslinking; consequently, syringyl-rich lignins are more linear compared to their guaiacyl-rich counterparts (Table 2) [7]. Lignin polymer is extended by the combination of monomeric radicals through cross-coupling reactions resulting in a linear molecule. Branching takes place through sequential nucleophilic attacks involving alcohols or hydroxyl groups on the benzyl carbon of a quinone methide (Figure 4). The structures of several important covalent bonds found in lignin are shown in Figure 5.

The biosynthesis of lignin is extremely important for understanding its post-synthetic fate in the cell wall of wood which in turn directly affects deconstruction strategies for lignocellulosic biofuel production. To illustrate, grasses can be delignified with greater ease than lignin from dicotyledons such as angiosperms and gymnosperms. This difference has been attributed to the high levels of *p*-coumaryl alcohol in grass lignin [12]. The mechanism of *in planta* formation of the lignin polymer has been studied *in vitro* using tissue culture model systems [16,30]. The advantage of such a system is that it eliminates the need for harsh chemical treatments for isolating lignin which might alter the structure. However, cell/tissue culture lignin might not be representative of the lignin occurring in natural wood.

A second model system to study monolignol polymerization is through the generation of dehydrogenation polymers (DHPs) primarily from the oxidation of coniferyl alcohol, most frequently catalyzed by peroxidase in the presence of H_2_O_2_. Two different types of crosslinkings were employed in generating the DHPs. The first, namely, ‘Zutropfverfahren’ or ‘Zutropf’ or Z_T_ DHP, is formed by adding the monolignol slowly and continuously to a reaction mixture resulting in the formation of “end-wise” polymers that resembled natural lignin by containing mostly β-O-4 bonds (Figure 5). The second type, ‘Zulaufverfahren’ or ‘Zulauf’ or Z_L_ DHPs are formed by bulk polymerization where all components (monolignol, H_2_O_2_, peroxidase) are mixed simultaneously, resulting in a larger proportion of β-β and β-5 linkages (Figure 5) [31,61]. Such model systems suggest that the type of lignin polymers in nature may be regulated by the availability, type, rate of supply of the precursors, and other factors listed above [15,37]. For example, bulk polymerization might take place in the middle lamella or the primary wall (more C-C bonds linking the monolignols) resulting in a highly branched polymer. Slow, end-wise polymerization occurring in the secondary walls results in predominantly β-O-4 coupling, yielding a more linear lignin polymer [62]. A mathematical model was published predicting different bond type frequencies and the number and abundance of different types of polymers that are formed [63]. The dehydrodimerization of coniferyl alcohol results in three different products with coupling being favored at the β-position resulting in β-β dimer (pinoresinol) (Figure 1), β-O-4- (β-ether) and β-5-dimers (phenylcoumaran) (Figure 5). Sinapyl alcohol dimerization results in only two products, namely, β-β dimer (syringaresinol) and β-O-4 dimer (β-ether) (Figure 5). The phenylcoumaran derivative is not formed since the crosslinking site is blocked by sinapyl’s second methoxy group. The β-coupling is favored with sinapyl alcohol compared to coniferyl alcohol [5]. Thus, the DHPs are a useful model for mimicking xylogenesis, directing the post-coupling events of the lignin polymer and its interactions with the polysaccharide components of the wood wall. 

Bond specificity in lignins is complex and complicated. The end-wise cross coupling of a monolignol radical to the free phenolic (hydroxyl) group of a lignin oligomer results in eventually extending it into the fully formed, β-linked polymeric macromolecule [6]. Limited availability of monolignol radicals, either by controlling their rate of availability or their *in situ* concentration, will favor cross-coupling reactions leading to the preferential formation of β-O-4 bonds. When a monolignol radical is close to a lignin chain that has not been radicalized, it is possible for the monolignol radical to transfer its higher oxidation state to the lignin chain. The reduced monolignol can then undergo a second round of oxidation by a cell wall-bound peroxidase or laccase and complete the cycle by coupling to the now radicalized lignin chain [28,31,37]. Alternately, RSMs might be involved in further radical generation and for maintaining lignin polymer growth [37,45]. 

Coniferyl alcohol reacting with a guaiacyl unit of lignin will result in G-β-O-4-G and G-β-5-G linkages whereas cross-coupling of two guaiacyl units from two different lignin chains yields G-5-5-G and G-4-O-5-G bonds. On the other hand, coniferyl alcohol reacting with a syringyl unit results in the formation of only G-β-O-4-S and not G-β-5-S, due to the presence of a methoxy substituent on the coniferyl alcohol at the 5-position. By contrast, coupling of a guaiacyl unit from one lignin chain with a syringyl unit of a different lignin chain will result in S-4-O-5-G bond but not the 5-5 bond. Finally, two syringyl units will not couple to each other [15]. Coupling of two lignin chains is a relatively rare event in syringyl/guaiacyl lignin but is encountered more frequently in guaiacyl lignin where 5-5 linkages may be present in amounts of ~5% of all linkages [27]. The ratio of syringyl-to-guaiacyl units in lignin determines the extent of crosslinking in the lignin polymer. Increased guaiacyl content leads to highly crosslinked lignin due to the presence of a larger number of biphenyl and C-C linkages. On the other hand, high levels of syringyl units yield lignins that are less crosslinked, more linear and connected by labile ether bonds at the 4-hydroxyl position (Table 2) [32]. The most labile bond is the benzylic ether, as it can be easily oxidized to a ketone with an increase in conjugation.

### 4.5. Quinone Methides

Quinone methides (Figure 4) are intermediates generated by radical coupling during monolignol polymerization to give rise to the lignin polymer [64]. Water addition results in β-O-4 linkage and phenol to the β-5 product [7]. As mentioned above, quinone methides play a role in introducing branching elements to the lignin structure through a nucleophilic attack involving alcohols or hydroxyl groups on the benzylic carbon. The β-ethers exist as two distinct isomers, ‘*erythro*’ and ‘*threo*’ arising from the addition of water molecule to one face of the quinone methide or the other. Each isomer has two enantiomers each possessing different physicochemical properties [5]. Acidic conditions (pH ≤ 5) favor the reaction of quinone methides with water to form a linear lignin chain. Less acidic conditions favor the reaction of quinone methide intermediates with monolignols to result in lignins that are more branched. Benzyl ester or benzyl ether linkages are predominantly formed under hydrophobic reaction conditions, a situation that is encountered most commonly during the final stages of lignification where the hydrophobic lignin polymer effectively excludes water from the cell wall [62]. Benzyl ester and benzyl ether linkages are also involved in linking the lignin to carbohydrates to form the lignin-carbohydrate complexes (LCCs), discussed elsewhere in this review.

### 4.6. Lignin Formation and Structure

Lignin is a complex molecule linked in a 3*D* network [36] and further linked to polysaccharides and perhaps even proteins. Ralph *et al.* [7] have suggested a “useful elegance in chaotic processes” of combinatorial chemistry of lignin synthesis that makes the polymer resistant to degradation which is critical considering the metabolic penalty that had to be paid for its synthesis [19,24]. There is scope for much research in the areas concerning the degree of randomness or the level of ordering in lignin formation and the polymer’s crystalline nature [21,65]. Supramolecular self-assembly processes may be involved in bestowing order to lignin structure. Lignin’s structural plasticity and its lack of regularity may work to the plant’s advantage by serving as a defense against pathogens or attack by enzymes secreted by invading microorganisms. The irregularity of the lignin structure requires a complicated evolutionary pathway in order to generate a single enzyme that is capable of recognizing and breaking all the various types of linkages found in lignin (Figure 5) [31]. Nature has circumvented this difficulty by endowing microorganisms with a battery of redox and hydrolytic enzymes that work in concert to degrade LCCs including the relatively easily oxidized benzylic ether linkages. 

Lignification is a complicated process [37] contributing to the remarkable ability of plants to survive a wide variety of biotic and abiotic stresses [30]. There has been little research done so far on the effects on lignin formation consequent to these stressors [30]. Plants adapt to significant changes in monolignol supply arising from natural causes or due to artificially introduced genetic lesions. The monolignol concentrations also differ widely (section 3) leading to gross or subtle changes to lignin chemical structure [66,67]. Once monolignol radicals are formed, it becomes difficult to predict or control the polymerization process. The differences between the structures of the DHPs and *in vivo* lignin illustrate the complexities of polymerization [68]. All these observations point to a high level of metabolic plasticity with regard to lignin biosynthesis which is to the plant’s advantage [22,37], but is also a major barrier to cost-effective lignocellulosic biofuels.

## 5. Lignans and Other Compounds

Lignans (Figure 1) are secondary metabolites that are also derived from phenylpropanoid units by dehydrodimerization, similar to lignin [69]. Lignans are distributed in various parts including roots, stems, leaves, seeds and fruits. Flax is one of the richest sources of lignans. Lignans are present at elevated levels in plants with high fiber content such as wheat, oats, beans, lentils and broccoli [69,70]. The polymerization mechanism for generating lignans is similar to lignin and involves the coupling of monolignol radicals generated by peroxidase and/or laccase catalysis. There is the one electron oxidation of monolignol resulting in the formation of the free radical resonance stabilized structures (Figure 4), finally resulting in oxidative coupling. The subunits of lignans are also made up of hydroxycinnamyl alcohols (mostly coniferyl alcohol) [32]. However unlike lignins, lignans are optically active due to stereospecific crosslinking [70]. Lignans undergo additional reactions following dimerization, giving rise to thousands of different phenolic compounds. Most lignans are dimeric in contrast to the polymeric lignins and correspondingly, the molecular mass of lignans is smaller compared to lignin. Several lignans are derived from 8-8' phenoxy radical coupling in addition to 8-5' and 8-O-4 linkages. The physiological function of lignans is speculative at this time. They are believed to play a role in defending the plants against pathogens or assist in plant growth and development [69]. There is a great deal of interest in lignans due to their physiological and pharmacological roles in human health and disease. Lignans display anti-tumor, anti-cancer, anti-inflammatory, antioxidative, antimicrobial, antiviral and immunosuppressive activities [69,70]. 

Monolignols also serve as intermediaries for the biosynthesis of other aromatic compounds such as vanillin and eugenol (Figure 1), the latter being responsible for the characteristic scent of basil [71]. Another class of related phenyl compounds is the flavanoids. Flavanoids comprise of thousands of molecularly related compounds found in higher plants. The pathway for flavanoid production involves *p*-coumaroyl-CoA of the phenylpropanoid pathway (Figure 3). Flavanoids include flavones, isoflavanoids, flavonols, flavandiols, anthocyanins (plant pigments), *etc.* [32]. Readers may consult the references cited in [32] for more information.

## 6. Lignin-Protein Interactions?

The plant cell wall is mainly composed of polysaccharides and lignin, but does contain small amounts of protein. Lignin deposition occurs in a scaffold composed largely of polysaccharides and some fraction of proteins as well. There are several hundred different types of proteins in the cell wall. A majority are glycoproteins exemplified by the hydroxyproline-rich glycoproteins (HRGP), proline-rich proteins (PRP) and glycine-rich proteins (GRP). The majority of the glycoproteins contain arabinogalactans (AGP) [72]. Extensin, a well characterized cell wall glycoprotein, and PRPs play a protective role in both mono- and di-cotyledons. These structural proteins are rich in basic amino acids such as lysine and amino acids such as hydroxyproline, proline and tyrosine. Several enzymes such as carbohydrases, oxidoreductases and proteases might also be associated with the plant wall [73]. It is likely that covalent and non-covalent complexes are formed between lignins and proteins. As far back as 1959, lignin-interactions were shown to inhibit lysozyme’s hydrolytic activity [74]. Compared to lignin-carbohydrate complexes (LCCs), lignin-protein interactions have not been investigated in depth.

There are important reasons for studying lignin-protein interactions. Aromatic residues of cell wall proteins were suggested as anchor sites for monolignols for enabling lignin polymer growth [28]. Ultraviolet (UV) analysis was reported to overestimate lignin content by 8-fold due to protein contamination [75] and could lead to mis-directed deconstruction strategies. Protein contamination will also result in inaccurate estimates of lignin by gravimetric techniques. The identification of lignin (or cell wall)-bound enzymes such as β-glucosidase capable of hydrolyzing coniferin and syringin might provide evidence of a role for phenylpropanoid glycosides in monolignol transport. One of the main reasons for the lignin barrier properties is the non-productive adsorption and inactivation of enzymes such as cellulases and β-glucosidases on the hydrophobic lignin surface [76,77,78]. The type of biomass pretreatments are also influenced by the affinity of cellulases and β-glucosidases for lignin and consequently their catalytic efficiencies [79]. 

A roadmap for conducting lignin-protein interactions was published by Tu *et al.* [80] during a systematic study of cellulase adsorption on Lodgepole pine lignin. An understanding of lignin-protein interactions might be the key to designing enzymes that bind with low affinity for lignin and escape inactivation. Weak lignin-binding enzymes were designed in order to improve the hydrolysis of lignocellulosics [81,82,83,84]. These are promising strategies for deconstructing biomass. Analyses of lignin-protein interactions will enable the down-selection of recombinant cellulases or β-glucosidases with poor affinity for lignin. Conversely, it might be possible design carbohydrases that are highly active despite the presence of lignin or (counter-intuitively) even display enhanced activity in (or due to) the presence of lignin. Such strategies might enable the circumvention of the lignin barrier or even bypass deconstruction efforts altogether. If carbohydrases can be designed to function efficiently even in the presence of lignin, then the lignin barrier does not exist. It should be noted that the lignin-protein interactions described in this section are not the same as the lignin-dirigent protein discussed above (section 4d). Lignin-protein interactions will enable the development of novel passivation agents or lignin blocking proteins such as gluten [85] or polypeptides that minimize or eliminate the non-productive interactions between cabohydrases and lignin. For example, lignin pre-treatment with bovine serum albumin (BSA) reduced the non-productive adsorption of cellulase and β-glucosidase on lignin, enhanced biomass conversion, and resulted in higher glucose yield [86,87,88]. 

The barrier effects of lignin might be partially related to its protein precipitating property due to the formation of hydrogen bonds between the lignin hydroxyl goups and the protein carboxyl groups. Such lignin-protein interactions could protect plant proteins from microbial degradation [89]. Identification of lignin binding domains (amino acid sequences or motifs) in proteins will enable the design of catalytically efficient recombinant enzymes (cellulase, β-glucosidase) lacking such domains. Such recombinant enzymes might be able to hydrolyze lignocellulosic biomass directly without prior pretreatment for removing the lignin. 

Intriguing data have been published hinting at lignin-protein interactions in the plant cell wall. For example, in the walls of the green alga *Chlamydomonas reinhardtii*, cell wall proteins are rendered insoluble by the production of H_2_O_2_ and peroxidase [90]. Interestingly, the production of H_2_O_2_ and peroxidase are also linked to lignin formation/deposition. Could the two events be connected? Extensin-like structural proteins were reported to be expressed in response to plant wounding, attack by pathogens, or other types of stressors [91]. Similarly, PRPs were found to become rapidly insolubilized in the cell walls of soybean cultures as a result of oxidative crosslinking [92]. These same conditions also trigger plants to produce *p*-coumaryl alcohol-enriched lignin at such sites, raising the possibility that lignin and proteins may become linked. Proteins are present in the cell wall before and during lignification and continue to remain as a structural element of the wood walls suggesting a role for proteins in xylem differentiation [92]. The middle lamella is rich in HRGPs and this region is also the most lignified zone of the plant wall. Indeed, the glycine rich protein GRP1.8 was deposited within the lignified rings of protoxylem [93]. Both HRGPs and GRPs were proposed to be associated with lignin and even act as focal points for lignin polymerization [94]. The lysine-rich extensin formed a positively charged scaffold that bound to the negatively charged pectin in order to create a matrix for the deposition of cell wall components including lignin [95]. Dill *et al.* [96] concluded that the nitrogen content of wood comes from proteins, some of which might be bound to the lignin. However, lignin-protein covalent bonds have not yet been proven definitively [97,98].

Whitmore conducted some of the early studies on lignin-protein interactions using tissue culture models and *in vitro* crosslinking experiments. DHPs (formed using ^14^C-labeled coniferyl alcohol, H_2_O_2_ and peroxidase) strongly bound to BSA, gelatin and synthetic polyhydroxy proline polymer [99]. *Pinus elliotti* cell wall preparations incubated with H_2_O_2_ and coniferyl alcohol also resulted in the formation of lignin that bound to a hydroxyproline-containing protein, speculated to be extensin [100]. Whitmore extended this work by showing that cell walls washed with detergent and 2M NaCl, increased its lignin weight fraction following incubation with coniferyl alcohol and H_2_O_2_, suggesting that cell-wall bound peroxidase was catalyzing the reaction. Furthermore, the lignin appeared to be covalently bound to a cell wall protein containing hydroxyproline. Whitmore suggested that polymerizing lignin was covalently coupled to cell wall structural proteins and that the occurrence of lignoproteins was consistent with the fungi degrading the lignin not only to access the cellulose but also the protein-bound nitrogen [101]. 

Tyrosine residues were implicated in the covalent crosslinking of horseradish peroxidase to DHPs of coniferyl alcohol catalyzed by the enzyme itself in the presence of H_2_O_2_. The crosslinked peroxidase was reported to be enzymatically active and a similar mechanism was proposed for lignification *in planta* [102]. Similar crosslinking of peroxidase to lignin through tyrosine-lignin bonds was also reported by Morimoto *et al.* [103]. Elicitor treatment or mechanical wounding of plants caused the insolubilization of 36 kDa peroxidase due to covalent crosslinking to lignin in the presence of H_2_O_2_, with the reaction being catalyzed by the enzyme itself, presumably involving its own tyrosine residues [103]. The authors concluded that the peroxidase might have a structural protein-like function in the cell wall in addition to its enzymatic function. Polylysine and polylysine/polytyrosine copolymer were covalently crosslinked to peroxidase-catalyzed coniferyl alcohol DHPs. The copolymers were crosslinked to a higher level relative to polylysine. Trypsin treatment released the synthetic polypeptides from the DHP. It was concluded that tyrosine residues enhanced protein crosslinking to lignin [104]. The role of tyrosine residues in protein crosslinking to lignin was extended to incorporate tyrosine-rich peptides into poplar lignin using transgene technology. The protein bonds were then cleaved using proteases in order to improve the saccharification efficiency [105]. 

It is clear from the foregoing that there are important reasons to focus research efforts into probing lignin-protein interactions. Lignins and proteins have been shown to interact through non-covalent and possibly covalent bonds. Alternate strategies for biomass deconstruction could be developed through a study of lignin-protein interactions. However, conclusive evidence for lignin-protein interactions *in planta* will require the isolation of lignoproteins and characterization of the covalent and/or noncovalent bonds holding the two polymers together. This includes isolating a lignin fragment covalently bound to a protein or a peptide or conversely a peptide fragment covalently bound to lignin. Such convincing evidence is not yet available leaving the physiological role (if any) for lignin-protein interactions unclear.

## 7. Lignin-Carbohydrate Complex (LCC)

In contrast to lignin-protein interactions, lignin-carbohydrate interactions are well documented. Indeed, there may be no “pure lignin” due to its tight association with cell wall polysaccharides. Bjorkman was the first to label these as “lignin-carbohydrate complexes” (LCCs) [cited in 106]. There is convincing evidence for covalent and non-covalent bonds holding lignins and carbohydrates together in the plant cell wall. Nevertheless, due to the complex nature of the cell wall and lignin, there are still many aspects of lignin-carbohydrate interactions that require further study. For instance, the polysaccharides are hydrophilic, whereas the lignin is hydrophobic and hence thermodynamically mismatched when the two are combined, resulting in phase separation, not unlike an oil-and-water mixture. Nevertheless, evidence suggests that lignin polymerization takes place in an aqueous carbohydrate matrix with the slow elimination of water until the environment becomes hydrophobic and a supramolecular LCC is formed [28]. Therefore, understanding the supramolecular complex is essential for biomass deconstruction. Since this review focuses on lignin, polysaccharides are only briefly described below. Further information regarding plant carbohydrates may be found in [107].

### 7.1. Major Plant Polysaccharides

**Figure 6:**
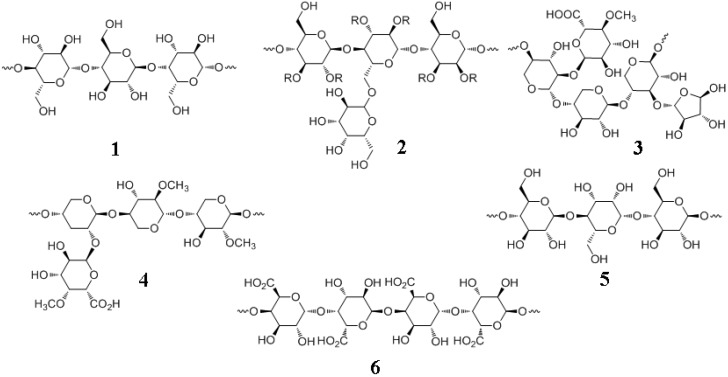
Important plant polysaccharides. **1**, cellulose, β-1,4-D-glucose (linear); **2**, softwood galactoglucomannan, β-D-1,4-glucose-mannose (linear) and α-D-1,6-galactose (branch); **3**, softwood xylan, arabinoglucuronoxylan, β-1,4-xylose (linear) and C_2_-4-*O*-methyl-α-D-glucuronic acid, C_3_-α-L-arabinose (branches); **4**, hardwood xylan, glucuronoxylan, 1,4-β-D-xylose (linear) and α-1,2-4-*O*-methyl-α-D-glucuronic acid (branch); **5**, hardwood glucomannan, β-D-glucose and β-D-mannose are alternately in β-1,4 linkages (linear); **6**, pectin, poly-α-1,4-D-galacturonic acid.

These include cellulose, hemicellulose (xylans, mannans), pectins, amylopectin, and amylose (Figure 6). Among these, amylose and amylopectin are storage forms of glucose whereas the other polysaccharides are structural units of the plant cell wall.

Lignocellulose, the desired component of biomass from a biofuels perspective, represents about one-half of all photosynthetically generated matter [108]. There are two main types of polysaccharides in the LCCs: cellulose and heteropolysaccharide (“hemicellulose”). Cellulose is a linear, crystalline homopolymer made up of D-glucose ranging from 8,000 to 15,000 residues per chain that are linked by β-1,4 glycosidic bonds (Figure 6) [12]. [This is in contrast to the storage form of plant glucose, namely starch, composed of amylose (*Mr,* 5 to 500 *kDa*), a linear polymer of D-glucose units linked together by α-1,4-bonds and amylopectin (*Mr*, up to one *MDa*), a α-1,6 branched form of D-glucose units held together by α-1,4-bonds]. A dimer of two glucose units linked by β-1,4 bond is known as cellobiose, which is a building block of cellulose and a product of cellulose hydrolysis by cellulase. Cellobiose is a strong inhibitor of cellulase (product inhibition). β-Glucosidases hydrolyze cellobiose, preventing the accumulation of the exoglucanase-inhibiting disaccharide. Cellobiose units linked together form the elemental fibrils which transform into cellulose microfibrils (composed of elemental microfibrils) and finally into cellulose fiber (composed of microfibrils) which is held together by hydrogen bonds and van der Waals forces. 

Hemicellulose is a misnomer since it is not related to cellulose. Hemicelluloses are better referred to as “heteropolysaccharides” or “crosslinking glycans” [109]. However, the term hemicellulose is widely used in the literature and is therefore retained here. Hemicelluloses are branched, heterogenous polysaccharides of shorter lengths compared to cellulose (about 500 to 3,000 residues) and composed of pentose (five carbons) and hexose (six carbons) sugars such as glucose, mannose, galactose, rhamnose, arabinose, xylose, 4-*O*-methyl-glucuronic acid, galacturonic acid and glucuronic acid. These sugars are linked by β-1,4 (predominantly) and β-1,3 (minor) bonds. The two main hemicellulose types are mannans and xylans (Figure 6). Mannans are composed of partially acetylated poly(β-1,4-D-mannose). Xylans are composed of partially acetylated poly(β-1,4-D-xylose). Xylans possess branch points of L-arabinose and 4-*O*-methylglucuronic acid. The dominant hemicellulose of softwoods is mannan whereas in hardwoods it is xylan.

Another important polysaccharide related to lignin deposition in the cell wall is pectin (Figure 6). Pectins are poly(α-1,4-D-galacturonic acid). The carboxyl groups of galacturonic acid are methyl esterified to varying extents. Therefore, pectins are copolymers of galacturonic acid and the methyl ester of galacturonic acid. Certain pectins also contain branched arabinans and linear galactans. Pectins are mostly found in the intercellular spaces as viscous gels, due to the action of Ca^2+^ ions. Elsewhere in this review, we will discuss in greater detail, pectin-lignin interactions taking place in the cell wall.

### 7.2. Mechanisms for Lignin-Carbohydrate Complex (LCC) Formation

As mentioned above, the main difficulty with studying lignin is its isolation in native, unaltered form. This difficulty extends to LCCs as well. Often the chemicals used and the pulping conditions (such as the Kraft process of heating wood chips in ~1M NaOH and ~0.2M Na_2_S for several hours at 150 to 180 °C) can create artificial linkages/bonds between lignins and polysaccharides. Thus, it is hard to determine authentic lignin-carbohydrate bonds occurring *in planta* relative to bonds arising from LCC isolation and/or processing conditions. More work is also required to identify enzymes that specifically recognize and cleave the bonds between lignin and hemicellulose. For example, the LCCs are degraded by the wood decaying actions of fungi and the microbes in termite hindgut. However, it was suggested that there are no enzymes specifically capable of cleaving the bonds in the LCCs. This was attributed to the low frequency of such bonds occurring in nature and the heterogenous character of these bonds [110]. Microorganisms employ a battery of enzymes such as cellulases, hemicellulases, peroxidases, laccases, esterases and oxidases that act in concert to solubilize the LCCs.

**Figure 7:**
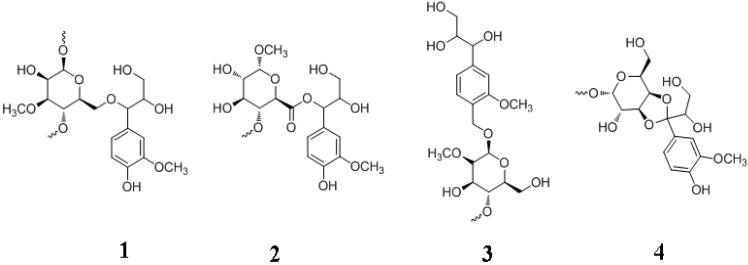
LCC Bonds. **1**, benzyl ether; **2**, benzyl ester; **3**, phenyl glycoside; **4**, acetal.

Four major types of covalent linkages were proposed to exist between lignin and carbohydrates. These are the benzyl ether, benzyl ester, phenylglycoside and acetal bonds (Figure 7) [111]. A qunione methide intermediate (Figure 4) is invoked where the electrophilic α-carbon is attacked especially under hydrophobic conditions to react with an alcoholic, phenolic, or carboxyl group, and giving rise to benzyl alcohols, esters and ethers. For example, *p*-coumaric and ferulic acid subunits in lignin might participate in benzyl ester and ether linkages with hemicellulose sugars. Benzyl esters are alkali labile whereas benzyl ether, acetal and phenylglycoside bonds are relatively alkali stable. With benzyl ethers, the α-hydroxyl group of lignin is connected to the hydroxyl group of carbohydrates. In benzyl esters, the α-hydroxyl group is linked to the carboxyl group of a glucuronic residue in a xylan. In the case of phenylglycosides, the alcoholic or phenolic hydroxyl group of lignin is linked to a mono- or a polysaccharide. Coniferin and syringin are examples of compounds containing the phenylglycosidic linkage. Finally, the acetal bond involves two hydroxyl groups of a polysacchride linked to lignin. The reaction of an acetal group with acid will produce hydroxyl and carbonyl groups. Lignin exists in a complex with hemicellulose through such covalent and other non-covalent bonds. Lignin is bound to cellulose only through non-covalent bonds [109,111]. 

Lignin-hemicellulose complex surrounds the cellulose with which it is bound through extensive hydrogen bonding to form a supramolecular structure that protects the cellulose and is the reason for biomass recalcitrance. The complex supramolecular structures of lignocellulose make it difficult to elucidate its physicochemical properties in the plant cell wall. However, *in vitro* studies have shown that the structure of lignin polymer is controlled by physicochemical conditions of the polysaccharide matrix in which lignin polymerization and deposition take place [112].

### 7.3. Mimicking LCCs

Due the crucial importance of LCCs in delignification and biomass deconstruction, it is useful to summarize a few *in vitro* studies of these complexes. An understanding of how LCCs are formed might enable techniques for breaking down the LCCs. Since monolignol polymerization and lignin deposition take place on carbohydrate matrices, information relevant to LCCs in plants might be obtained by carrying out monolignol polymerization *in vitro* in the presence of hemicellulose or pectin. Computational approaches will also assist in comprehending the nature of LCCs. The extreme complexity and variability of the lignified plant cell walls make *in vitro* model assemblies a useful tool to study LCCs and their impact upon saccharification efficiency as well as to understand cell wall assembly. *In vitro* experiments permit control of the reaction composition with respect to monolignol type and concentration, the presence (or absence) of specific carbohydrates to form the artificial matrix, the choice and combination of polymerizing enzymes (laccase, peroxidase) and the polymerization conditions. 

The type of polymerization had a profound effect upon the covalent or non-covalent bonds between DHPs and polysaccharides. For example, when coniferyl alcohol was polymerized by horseradish peroxidase and H_2_O_2_ in the presence of xylans, covalent benzylether linkages were formed between ‘Zutropf’ DHPs and the polysaccharide, involving the 5-hydroxyl of L-arabinose and the α-carbon of DHP-coniferyl alcohol. However, when ‘Zulauf’ technique was adopted, only noncovalent bonds were observed between xylan and DHP. The resulting xylan-DHP supramolecular nanocomposites were resistant to endoxylanase digestion [106,113,114,115]. Uraki *et al.* [116] reported that the hemicelluloses xylan and mannan adsorbed the monolignols non-covalently through hydrophobic forces. Coniferyl and sinapyl alcohols were adsorbed to a greater extent than their corresponding glycosides, coniferin and syringin. Physiologically, this is useful since cell wall associated β-glucosidases might release the monolignol which then undergoes radical formation and polymerization into the growing lignin chain embedded in a hemicellulose matrix. The affinity of monolignols for the hemicellulose could assist in the polymerization process. 

Structural and phase-dependent changes were also reported depending on the polymerization conditions (‘Zulauf” *versus* ‘Zutropf’) as well as the polysaccharide matrix [112,117]. Thus, when Zutropf DHPs of coniferyl alcohol were formed using peroxidase/H_2_O_2_ in the absence of carbohydrates, the synthetic lignin precipitated from the reaction mixture. However, when polymerization was carried out in the presence of pectin, the reaction mixture formed a colloidal suspension that remained stable for several months. The authors concluded that the latter type of synthetic lignin mimicked lignin formation in plants (117). 

In conclusion, we are developing a greater understanding of LCCs and their role in preventing cellulose fermentation; this knowledge is essential for launching cost-effective lignocellulosic biofuels.

## 8. Supramolecular Self Assembly

### 8.1. Xylogenesis

Cell wall formation involves supramolecular self-assembly, a process that is foundational for the generation of functional nanoparticles [118,119]. Lignified cell wall formation (xylogenesis) begins with the deposition of pectin, hemicellulose, cellulose, and ends with lignin deposition into this polysaccharide matrix [117]. These wall components are physically and chemically bound together in a supramolecular architecture. Grabber [62] outlined several models for studying the supramolecular cell wall complex: (1) study isolated cell walls; (2) study lignification in plant cell/tissue culture models; (3) study *in vitro* DHP formation in the presence of cell wall components such as xylans or pectins. Noncovalent hydrophobic interactions result in the formation of supramolecular structures of the cell wall. During xylogenesis, the local environment is changed from hydrophilic to hydrophobic by the gradual elimination of water. Indeed, Inomata *et al.* [120] confirmed the removal of water during plant cell wall lignification *in vivo*. Dehydration enhances the local polysaccharide concentration and also reduces water attack on the quinone methide intermediate (Figure 4), enabling hemicellulose reactions to generate covalent benzyl esters and ethers of the LCCs (Figure 7). Consequently, the concentrations of lignin and LCCs probably track in the same direction [115]. Nucleation sites have been proposed for promoting lignin polymerization [28]. Ferulic acid (Figure 1) derivatives [121] or aromatic amino acids (tyrosine) in proteins have been suggested to anchor the monolignols to enable polymer growth [28]. The details are unknown of how a growing plant coordinates the synthesis and deposition of hydrophobic phenolic and hydrophilic carbohydrate biopolymers to result in a supramolecular architecture of the cell wall [12].

### 8.2. Cell Wall Formation

**Figure 8:**
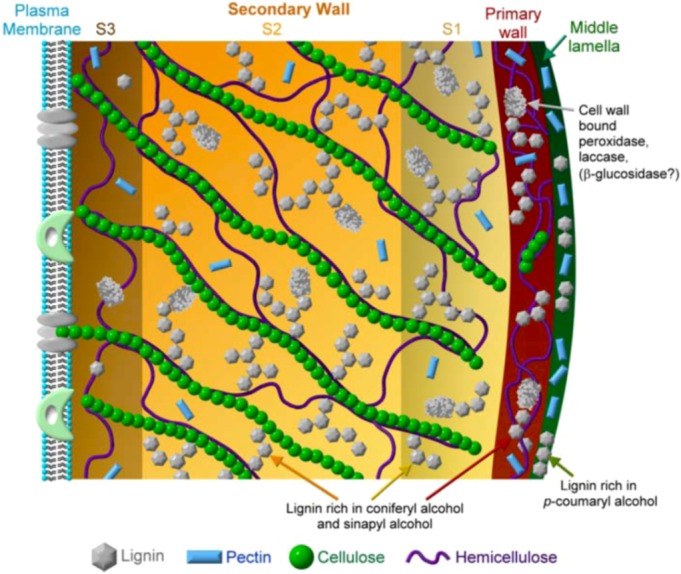
Illustration of a plant cell wall. The various features of the plant cell wall described above are shown including the relative thickness of the various layers and the relative abundance and specific localization of the various cell wall components, such as pectin, cellulose, hemicellulose, lignin and protein. The relative contributions of the three major monlignols to the lignin in the various layers are also indicated. The cell wall-bound enzymes might participate in the various steps of lignification.

The chemical, physical and biological properties of wood are determined not only by its components, *i.e.,* cellulose, hemicellulose, pectin, lignin and structural proteins, but also by their relative proportions. Plant cell walls range in thickness from 100 to 1,000 nm or more. Xylogenesis is associated with xylem thickening, reinforced by lignin, which is familiar as the “tree rings” of wood. The plant cell wall is interesting in that it grows from “outside in,” contrasting the usually “inside out” growth of cells and tissues. The outer layer of the middle lamella is deposited first. The middle lamella is enriched with pectin polysaccharides and serves to bind adjacent cells together. The primary cell wall, a thin stretchable layer of cellulose microfibrils linked to hemicellulose, is deposited next inside the middle lamella. The microfibrils range from 5 to 15 nm in diameter and are several microns long. The primary wall is composed of <10% of cellulose, but is abundant in hemicellulose and pectin along with structural proteins. Finally, the thick secondary wall, containing the vast majority (~95%) of the cellulose polysaccharides, is deposited inside the primary wall. The secondary wall is composed of the LCC of cellulose, hemicellulose, pectin and lignin (Figure 8). All plant cells have a middle lamella and a primary wall. Secondary walls are found in specialized tissues such as the xylem.

Flowering plants have “Type I” walls containing approximately equal proportions of cellulose and xyloglucans covalently crosslinked to pectin and structural proteins. Grasses and certain monocotyledons have “Type II” walls where the cellulose is linked to arabinoxylans. In contrast to Type I walls, the Type II walls of grasses have fewer structural proteins. Xylan is the major (non-cellulosic) polysaccharide in the secondary Type I wall whereas galactose-bearing polymers are more abundant in Type II walls. Lignin deposition is probably the final step in the differentiation of xylem secondary wall and takes place after most of the polysaccharides have already been deposited [122]. Hydrophilic pectin gels are found in the middle lamella and cell corners along with cellulose and hemicellulose where lignification is initiated [112,117]. 

Thus, lignification takes place in a carbohydrate matrix of the middle lamella and secondary walls. The lignin nanoparticles (10–70 nm) are probably scattered at first and then coalesce to form the lignin polymer, while simultaneously driving out water [117,120]. The secondary wall may be further distinguished by its constituent layers of S1 (outer), S2 (middle) and S3 (inner), where the cellulose microfibrils orient differently and the lignin composition is also varied (Figure 8). Lignin is rarely found in the S3 layer. Lignin in the middle lamella is enriched in *p*-coumaryl alcohol whereas coniferyl alcohol is largely targeted to the lignin of the primary and secondary wall layers of S1 and S2. Sinapyl alcohol is found in the fiber forming cell walls. The secondary wall is in contact with the plasma membrane that surrounds the cytoplasm [9,36]. The reader may consult reviews by Gorshkova *et al.* [123] and Boudet [124] for more details of the supramolecular self-assembly of the lignified wall.

### 8.3. Supramolecular Lignin

The supramolecular organization of lignin in the cell wall profoundly affects cost-effective lignocellulosic biofuel production and requires an interdisciplinary approach [124]. Studies of lignin are complicated by its complex composition, primary sequence, enzymatic and non-biochemical mechanisms for synthesis and also due to its propensity to self-associate as well as interact with carbohydrates and proteins to form supramolecular structures. At the polymerization stage, the distribution of the monolignols is non-deterministic due to the absence of biological control and the non-enzymatic, free radical reactions resulting in an unpredictable primary phenolic sequence. The molecular mass distributions of lignin depend significantly on the plant species and differ even among various parts of the same plant. It is likely that the supramolecular lignin organization varies depending on its specific location within a plant’s structure such as root, stem or leaves and their developmental stage along with other biotic and abiotic factors such as the plant species, the chemical environment under which the lignin polymer was formed, *etc.* [30]. Further contributing to difficulties are the self-associative behavior of some of the polysaccharides and the reciprocal influences of the carbohydrates and lignin on the self-assembly properties of each other. All these variations affect the chemical and mechanical properties of wood and hence the biomass processing that is critical in the effort to produce cost-effective lignocellulosic biofuels. These interactions and structures are briefly discussed beginning with the coneptually simpler self-interactions of lignin polymers and proceeding to the more complex lignin-carbohydrate supramolecular self-assemblies.

Lignin polymers showed self-assembly behavior in solution that was reversibly controlled by the polydispersity of the polymer and environmental factors such as the solvent type, pH, ionic strength and temperature [109,125,126]. Ultrafiltration and light scattering studies demonstrated large, stable self-assembled lignin complexes with decreasing pH and/or increasing polymer concentration. Protonation of phenolic hydroxyl groups of lignin resulting in the formation of highest occupied molecular orbital-lowest unoccupied molecular orbital (HOMO-LUMO) bonding of π-orbitals of the benzene rings was attributed to lignin self-assembly [127]. Lignin aggregates with a hydrodynamic radius of 60 nm were shown to be formed in dioxane-water mixture due to non-covalent electrostatic and van der Waals forces between the polar and apolar groups of the lignin polymer [128]. Increasing the temperature disrupted lignin self-assembly whereas lignin aggregation increased at 4 °C resulting in large molecular mass species that could be discriminated using size-exclusion chromatography (SEC). Furthermore, softwood lignins displayed a greater tendency for reversible self-assembly compared to hardwood lignins (Table 2) [129]. The effect of environmental factors such as ionic strength, pH, temperature, solvent, time and aging effects upon lignin self-assembly were studied by changes to the molecular mass using the light scattering techniques of multiangle laser light scattering (MALLS) and differential refractometry (DR) [130]. Lignin molecular weight distributions were related to guaiacyl and syringyl content and structural variations such as hardwood lignin being more linear than softwood lignin (Table 2). 

Using microscopy, Micic and colleagues published a series of papers [131,132,133,134,135,136] describing the supramolecular self-assembly of lignin in model systems of *in vitro* DHPs and photopolymerization of coniferyl alcohol. They envisioned lignin at the nanoscale as being globular with elastic and visco-elastic properties due to intermolecular π-π interactions, hydrogen bonding and van der Waals forces amongst the macromolecular globules resulting in semi-ordered superstructures. Micic *et al.* postulated that *in vitro* polymerization of coniferyl alcohol formed a module of about 20 units that polymerized further into a supermodule (~500 units). A large number of supermodules spontaneously self-assembled to form clusters/flocks of globules. Gobules rearranged into colloidal assemblies or crystals. The size varied by 6 orders of magnitude for these various structures from the nanometer scale for the modules to fraction of a millimeter at the colloidal crystal stage. The rheological and nano-mechanical properties of the shell-like layered lignin were related to its structure and shape and played a key role in determining its physiological function by providing directionality within geometrically constrained spaces and thereby enhancing the rigidity and mechanical stiffness of the cell wall. Since natural lignin is achiral, lacking optical activity [55], and the synthetic DHPs were similar in these respects, it was concluded that the DHPs were a reasonable model system for *in planta* processes. The supramolecular self-assembly of lignin polymers complement its associative behavior with carbohydrate polymers, contributing to the difficulties in isolating or studying “pure lignin.”

### 8.4. Substratum Effects on Supramolecular Self-Assembly

Lignin polymerization occurs on a preformed carbohydrate matrix. Therefore, *in vitro* DHP studies might be more meaningful in the context of matrix effects influencing lignin supramolecular structure and conformation on the nanoscale [68,137]. The substratum becomes a scaffold upon which lignin deposition takes place and the scaffold composition naturally exerts pronounced effects upon lignification. Substratum molecules included cellulose, xylan, mannan, pectin composites, arabinoxylan, cyclodextrins and perhaps proteins. The chemical composition and concentration of the scaffold, environmental factors (pH, ionic strength, *etc.*), monolignol diversity and relative affinities, all cooperatively direct the lignification process. The substratum effects were confirmed by *in vitro* DHP formation upon relatively smooth, synthetic surfaces of graphite (hydrophobic), mica (partially hydrophilic) and glass (hydrophilic). The eventual shape of the synthetic lignin superstructures were controlled by substratum’s surface properties due to π orbital and its hydrophobic and/or hydrophilic nature and such effects might extend to *in planta* lignin formation [132,138]. 

**Figure 9:**
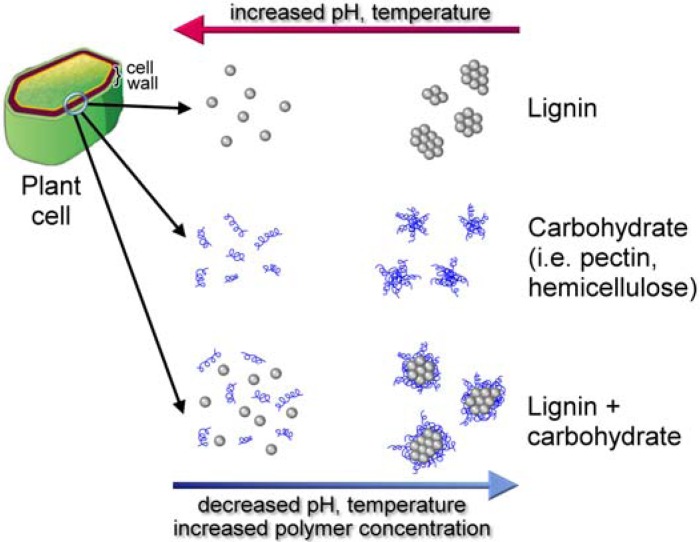
Schematic of supramolecular self-assembly of lignin and lignin-carbohydrates interactions.

Zulauf DHPs of coniferyl alcohol polymerized in the presence of cellulose were arranged in a single layer of 400 to 1,000 nm thickness consisting of 800 to 1,000 nm sized hexagonal structures unlike synthetic lignin formed in the absence of the matrix, supporting an important role for the carbohydrates during lignification [138]. A detailed analysis of matrix effects on DHPs was conducted by Barakat *et al.* [113] using guaiacyl and guaiacyl/syringyl monomers for DHPs in the presence of two different types of xylans: one that was enriched in ferulic acid and the other devoid of ferulic acid. The resulting nanoparticles were examined by transmission electron microscopy (TEM), SEC and MALLS. The ferulic acid-substituted xylan resulted in the formation larger and denser nanoparticles composed of xylan-DHP complex. Xylan self-associated strongly and also adsorbed strongly onto cellulose surfaces. The latter adsorption was promoted by cationic and hydrophobic substituents which enhanced the amphiphilic character of the hemicellulose [109]. Xylan also associated with lignin and lignin enhanced the self-aggregation of xylan [109,113]. All these properties might indeed guide lignification *in vivo* [113]. A simplified view of lignin-substratum interactions is shown in Figure 9.

### 8.5. Lignin Conformation

Studies using theoretical, microscopic and biophysical analyses have concluded that lignin displays, at least partially, an extended helical structure [131,138]. This observation recalls the other, perhaps more famous helical biopolymer, the DNA. However, the helical structure of lignin is quite unlike that of DNA, since there is neither sequence-specific complementarity of DNA nor are the bonds similar. Lignins have a high content of β-O-4 (alkyl aryl ether) bonds (Figure 5) resulting in the polymer adopting a spiral, quasi-helical conformation that is dependent on its syringyl content [139]. Therefore, one might expect guaiacyl-syringyl lignins of angiosperms to display more coiled spiral conformation with a greater longitudinal expansion relative to gymnosperm lignins, which have a higher content of β-β and β-5 bonds (Figure 5). On the other hand, several investigators have reported a spherical, globular or disk like conformation for technical lignin or synthetic DHP [131,132,133,134,135,136,140]. Deconvolution fluorescence spectroscopy of DHPs suggested a multi-layered structure for lignin [141]. 

Isolated and synthetic lignins were shown to have a high degree of flexibility enabling the polymer to adopt different stable or metastable conformations, along with changes to molecular size and volume due to ester bonds in the polymer. Noncovalent bonds are probably important in stabilizing the various conformations. Surface potential area isotherm calculations suggested that it was impossible to have the same conformational arrangement twice for these highly random biopolymers [134]. Hardwood lignins were reported to display a regularly branched star shaped topology, whilst softwood lignins are likely to exist as randomly branched structures [142]. The topology of grass lignins has not yet been clarified [142]. Natural lignin is thought to be overall a linear polymer. It is difficult to examine lignin *in planta* using X-ray or other imaging techniques [14]. Therefore, whether any of the aforementioned conformational transitions occur *in vivo* is speculative. Many solvents used for dissolving lignin (acetone-water, 9:1; dimethylformamide; dimethylsulfoxide; 2,4-dioxane-water, 9:1; 1M ammonium hydroxide) [140] or the Kraft process can profoundly alter lignin structure including scission of the alkali-labile bonds. Consequently, most of the information regarding lignin conformation are only an approximation of *in vivo* lignin.

### 8.6. Fractal Properties of Lignin

**Figure 10:**
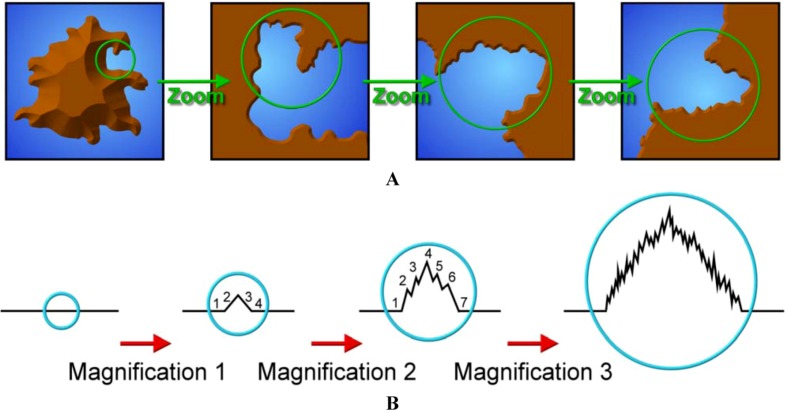
A. Schematic of a coastline demonstrating the concept of a natural fractal’s self-similarity. Note the similarity of contours at various levels of magnification. **B.** Schematic of a dimensional fractal. Notice the increase in *Df* with increasing levels of magnification (shown inside the circled areas).

Before discussing the fractal properties of lignin, it might be useful to provide a brief introduction to fractals. A detailed dissertation of fractals is beyond the scope of this review and the reader is referred to an excellent book on the topic [143]. Typically, objects are described according to Euclidean geometry, by their shapes such as lines, squares, circles, triangles, spheres, *etc.* Natural geometries are not so easily described by Euclidean geometry alone. Benoit Mandelbrot introduced the concept of fractal geometry to describe fractal objects, which are simply referred to as fractals [143]. An elementary description of a fractal is that it possesses the property of self-similarity under different degrees of magnification. In other words, the structural details seen at varying levels of magnification closely resemble its macroscopic profile; each small part of a fractal displays the same geometrical details as the whole fractal object (Figure 10A). 

It is important to note that natural fractals display only statistical self-“similarity” and are not *identical* under various levels of magnification. By contrast, computer generated (*i.e.*, mathematical) fractals are precise in their self-similarity at all scales of magnification. The fractal dimension (D_F_) is larger than the object’s topological dimension (D_T_). On the other hand, D_F_ is smaller the Euclidean dimension (D_E_) for the same object. These dimensions provide a quantitative assessment of the extent of self-similarity at various magnifications. A second property of a fractal is “scaling.” Features observed at higher levels of magnification are indeed smaller copies of the same features seen at lower power. Consequently, the lengths observed at higher magnification will be longer than those measured at lower magnification, as illustrated in Figure 10B. Scaling is defined as “the value measured for a property depends on the resolution at which it is measured” [143].

Lignin as a fractal object was first described by Ozol’-Kalnin *et al.* in 1986 (*Khim. Drevesiny* **1986,** *5*, 108; cited in reference 13). Since then, the fractal properties of lignin have been thoroughly reviewed [13]. In the case of lignin, the mass fractal dimension, D_F_, is a measure of the distribution of the structural features at varying levels of magnification. For lignin, D_F_ is calculated from the scaling indices of its hydrodynamic parameters such as intrinsic viscosity, diffusion coefficient, scaling index, sedimentation constant, *etc.* [142]. The conformational properties of a biopolymer such as lignin is governed by its primary structure based on the ionizable groups that in turn are dependent on the milieu properties of solvent type, ionic strength, pH and temperature. These conformational properties are manifested as rotational and frictional processes [13]. Consequently, the conformational and topological properties of lignin are studied using molecular hydrodynamic parameters [144]. Lignin has been described as having a globular conformation of ~20 nm in diameter that corresponded to an approximate molecular mass of 55 kDa for a single lignin polymer chain [138]. However, a globular object is not a fractal [13]. To reconcile the fractal properties of lignin, it is assumed that lignin adopts a coiled or helical conformation possessing fractal dimensions [131,141,145,146].

Lignin displays statistical self-similarity and scale invariance and thus qualifies as a fractal object. Non-crystalline nature of the chaotically crosslinked lignin polymer also favors a fractal structure at least over some scale range [13]. The aggregation of polymers can also be used to determine the fractal dimensions by measuring its hydrodynamic properties [13]. Fractal objects may be formed under conditions of diffusion-limited polymer aggregation. If the diffusion of monolignols to lignification sites plays a role in lignin biosynthesis, then fractal theory is useful for understanding lignin polymerization, macromolecular structure, and supramolecular organization [146]. As described above (section 8c), lignin displays self-aggregation property resulting in colloidal structures in solution. These particles have been shown to further self-associate to form fractal aggregates with size regimen of 100 to 2,000 nm as determined by TEM and small angle X-ray scattering (SAXS) techniques [14]. Scanning tunneling microscopy (STM) images of coniferyl alcohol DHPs determined the synthetic lignin’s fractal dimension as approximately 1.9. The synthetic polymer displayed fractal features of regularity at several levels of organization [146,147]. Softwood guaiacyl lignins especially have been suggested to possess randomly branched supramolecular structural organization with fractal characteristics based on deterministic chaos concept analysis [148]. A number of microscopic techniques including atomic force microscopy (AFM), STM, near field scanning optical microscopy (NSOM) and environmental scanning electron microscopy (ESEM) have been used to obtain images of model lignins that revealed highly ordered fractal structures. Fractal theoretical analyses were also used to document the structural regularity and organization of synthetic lignins [149].

Lignin polymeric organization has been described as multi-level with each level less complex than the one above. These levels begin with chemical complexity due to the different constituent monolignols, moving up to molecular complexity based on primary sequence, then to macromolecular level, progressively increasing in complexity at topological, supramolecular and ultrastructural levels. Theoretical analysis at higher levels of complexities involves the concepts of deterministic chaos, fractal theory and non-linear dynamics [13]. In conclusion, fractal analyses of lignin might facilitate a better understanding of not only the supramolecular organization but also help to clarify the biosynthetic and polymerization processes. This knowledge could enable the design of improved biomass deconstruction strategies in the difficult path towards cost-effective lignocellulosic biofuel.

### 8.7. Wood Attributes

Supramolecular organization is critical to understanding the properties of wood that in turn are essential not only for developing better deconstruction strategies but also important to the topics of biomimicry/biomimetics and bioinspired nanocomposites. For example, Barakat *et al.* [114] after a study of the supramolecular organization of xylan-DHPs suggested that such hybrid polymers might be useful in designing new nanoparticles. A biomimetic study of the *in vitro* collapse of pectin chains as a result of DHP formation in a pectin matrix was extrapolated to occur in living plants suggesting macromolecular pectin reorganization during lignification [117]. However, several aspects of the supramolecular organization of lignin interactions with cell wall components and the effects of biotic and abiotic stressors are poorly understood [30]. Even everyday phenomenon such as drying of wood is not well understood [150], even though it plays a critical role in lignocellulosic biofuels. Trees grow under hydration conditions (watery environment) with the xylem and phloem involved in the transport of nutrients and minerals from the roots to all parts of the tree. However, once a tree is cut down, it gets dehydrated leading to irreversible changes in supramolecular structures including cellulose aggregation and changes to the physico-chemical properties of the LCCs. The removal of water from a felled tree is affected by temperature and humidity. This dehydration modifies the wood’s mechanical and physical properties, which in turn influence bioconversion [150]. 

Wood is a natural composite of several different types of hydrophilic and hydrophobic polymers. Another area of interest in the study of wood is its use as a model system for the synthesis and characterization of artificial composites. Synthetic, multi-phase composites can and do fracture due to transition between phases and interfacial instability. A natural composite such as wood does not fracture in this manner and plants have solved the interfacial instability and incompatibility issues. Nature has evolved gradual transitioning techniques between incompatible regions such as the hydrophilic cellulose and the hydrophobic lignin. Understanding self-assembly processes in wood will enable the development of novel nanocomposites [151].

Stems or branches that grow in an angular fashion can be forced to grow in a normal way through the development of a particular type of wood known as reaction wood. Thus, reaction wood is the product of a stressing situation for the plant [30]. Reaction wood is of two types: a) tension wood and b) compression wood (Table 1). Plant signaling molecules such as auxin may be involved in the biosynthesis of reaction wood. Tension wood develops from *above* the angular growth, *pulling* the leaning stem/branch upwards. Compression wood on the other hand develops from *below* the leaning region *pushing* it upwards [30]. The physical, chemical and mechanical properties of the two types of reaction wood are different. For example, tension wood has lower levels of lignin compared to compression wood. Reaction wood is considered as a model for *in vivo* lignin biosynthesis [152].

## 9. Tools and Techniques

The use of established tools and techniques and the development of novel assays are crucial to support lignocellulosic biofuels development. Such tools and techniques also inform us on the structure and conformation of lignin and enable us to evaluate the various strategies for biomass dissolution and processing. The National Renewable Energy Laboratory (NREL) located in Golden, Colorado has developed Laboratory Analytical Procedures (LAP). The LAPs are periodically revised to accommodate new developments in the field in order to assist scientists engaged in biofuels research (www.nrel.gov). In this review, we have mentioned different absorption, fluorescence and light scattering techniques for the study of lignin. In this section, we will explore the various techniques for lignin and cell wall analyses in greater detail. 

Microscopy is intimately connected to plant cell imaging since the time of Robert Hooke (1665) when he observed the structure of cork under a microscope and coined the term “cell” for the first time to describe the basic unit of a tissue. Since then, great strides have been made in imaging plant cells and characterizing its ultrastructure. Recent developments include confocal microscopes that are capable of spectral and hyperspectral analyses for discriminating against plant autofluorescence [153,154]. Single- and two-photon confocal microscopy and deconvoluting software [141] have made plant cell imaging sophisticated and yield a vast quantity of information regarding cell structure [153]. High content screening (HCS) embraces diverse tools and disciplines such as optics (flow cytometry, fluorescence microscopy), chemistry, biology, high-throughput screening (HTS) and image and data analysis software. HCS is widely used for drug discovery in the pharmaceutical industry [155]. Using HCS, structural and functional evaluation of cells are carried out in spatially and temporally defined manner in order to monitor biochemical, genetic and morphological changes. Nevertheless, true HCS of plants is not yet a reality. The hydrophobicity of the plant wall and the lignin impermeability network, while advantageous to the plant, makes it difficult to design traditional antibody or labeled protein-based probes (such probes are compatible with animal cells).

For efficient biomass conversion, it is essential to understand the interplay between the various levels of plant organization beginning with the cells and ending with the entire plant. Scientists might take a page out of Nature by studying the various microorganisms that thrive on biomass. For example, chemical genetics, biotic or abiotic stressors [30] resulting in the up and/or down regulation of certain lignin biosynthetic or degrading enzymes might be studied using HCS in order to construct systems biology models of cell structure and function with the goal of mitigating biomass recalcitrance. Even simple staining techniques might provide useful information. The presence of aldehyde groups in lignin has been used to stain plant cells with phloroglucinol [22] and study lignin biodistribution [156]. However, the challenge of detecting cell wall changes in HTS format remains [2,124]. 

We have already commented on the difficulties of obtaining “pure lignin” and how chemical treatments (solvents or Kraft cooking with or without LiBr, a chaotropic salt, to assist in lignin solubilization) [106,140] could irreversibly modify lignin structure. Estimation of lignin content employs several different methods including acetyl bromide, Klason, and acid treatment or thioacidolysis [111,157]. Attempts at isolating lignin become destructive due to lignin’s tight association with cell wall polysaccharides and therefore non-representative of *in vivo* supramolecular organization. However, a study of lignin at macro- and micro-scopic scales is exceedingly important in view of the polymer’s barrier properties. In this context, SEC is a valuable tool for polyphenolic lignin isolation and purification [113,114]. 

Microscopy techniques such as Fourier transform infrared Raman (FTIR-Raman) microscopy, total internal reflection fluorescence microscopy (TIRF), AFM, TEM, NSOM, ESEM, STM, SAXS, scanning electron microscopy (SEM), field emission-SEM (FE-SEM), scanning transmission X-ray microscopy (STXM), X-ray absorption near edge spectroscopy (XANES), along with spectroscopic (ultraviolet, UV, visible, near-infrared, NIR) tools such as absorption and fluorescence, Raman spectroscopy in its various incarnations, X-ray diffraction, nuclear magnetic resonance (NMR), mass spectroscopy (MS), 2-D NMR, electron spin resonance (ESR), electron paramagnetic resonance (EPR) and the various light scattering techniques, collectively will advance our knowledge of lignin supramolecular organization and assembly into the plant cell wall [2,124,157]. 

Surface properties of the lignin biopolymer may be investigated using electron spectroscopy for chemical analysis (ESCA), a measurement that is also referred to as X-ray photoelectron spectroscopy (XPS). This is a surface-sensitive technique and provides information regarding lignin chemical composition. In addition to these techniques, methods for studying lignin’s rheological and mechanical properties must also be emphasized, as they too impact biomass dissolution. These properties include viscosity, elasticity, rigidity, dynamic contact angle measurements, and lignin’s ability to withstand turgor pressure *in planta*. Finally molecular modeling, theoretical calculations and computer simulation must be adopted in order to obtain a global view of lignin structure, ordering and supramolecular organization, beginning with the primary structure and sequence and how composition affects secondary and tertiary structures, and finally macroscopic assembly into the plant cell wall. The physico-chemical, structural, conformational and organizational features of macromolecular lignin *in vivo* are mostly unknown and there is ample scope for in-depth research on these topics. The launching of cost-effective lignocellulosic biofuels will depend upon such developments.

The techniques described above are labor intensive, require extensive sample preparation and are therefore neither rapid nor suitable for HTS. These techniques frequently involve large instruments that are not portable for on site measurements. Furthermore, sophisticated technologies, despite their inherent high sensitivity, are usually expensive. These technologies are frequently destructive in that the biomass analyzed also becomes unrecoverable. On site, *in situ*, label-free measurements of lignin compositional analyses are crucial for prompt implementation of smart deconstruction strategies for lignocellulosic biomass dissolution. Lignin composition, especially the content of coniferyl and sinapyl alcohols and their relative ratios, determine the ease of deconstruction. A survey of the lignin/cell wall literature revealed two or three technologies that are prominently used. These techniques included molecular spectroscopy/microscopy, Raman spectroscopy/microscopy, NMR and scanning probe microscopy. We will describe these tools for investigating lignin and its chemical composition and introduce some of the more popular techniques within the context of specific, referenced studies.

### 9.1. Raman Spectroscopy

Raman spectroscopy (Nobel Prize-winning work of Sir C.V. Raman [158]) and microscopy are non-destructive technologies that exploit a molecule’s vibrational and rotational modes and monochromatic light scattering properties. Raman spectrum is exquisitely sensitive to perturbations and is thus a sensitive signal for conformational changes. Since its discovery, Raman spectroscopy has evolved into one of the most widely used technology for molecular analyses of solid specimens. It is a non-invasive, label-free technique for plant image analyses [159]. Raman signatures of the phenolic monolignols will be registered at 1,600 cm^−1^ (aromatic), the carbonyl and unsaturated carbon double bonds at 1,735 cm^−1^ and 1,650 cm^−1^, respectively. Thus, chemical imaging by confocal Raman microscopy was used for the spatial distribution of cellulose and lignin in wood walls *in situ* [160]. A review by Agarwal [159] summarizes the use of Raman spectroscopy for plant cell imaging.

### 9.2. Molecular Spectroscopy

Fluorescence is perhaps the most widely used and highly sensitive technique for molecular analysis in solution with detection limits down to the single molecule level [161]. Autofluorescence is a natural phenomenon of plants due to the presence of certain lipids and chromophores such as chlorophyll, the green pigment of leaves and fluoresces at >600 nm wavelengths, when the sample is excited between 420 and 460 nm, requirng filters to block these excitation wavelengths and suppress background fluorescence in order to enable signal discrimination [153]. Plant cell wall fluorescence is strong and computational software must be used to distinguish amongst similar fluorophores [162]. Lignin contributes significantly to cell wall fluorescence over a wide range of excitation and emission wavelengths due to a variety of aromatic, aryl conjugated carbonyls, stilbene and ferulic acid structures (Figure 1) [157]. Autofluorescence of plant cell walls was used to track solubilization during ionic liquid-mediated deconstruction of switchgrass biomass without interference from lignin. This enabled the label-free visualization of cell wall dissolution [163]. Fluorescence was used to develop various types of “lignin sensors” and fluorescence was used to interrogate coniferyl alcohol and its oxidation by laccase and peroxidase in a HTS format [164]. 

Hatfield and Fukushima summarized the various techniques for isolating, characterizing and quantitating lignin [165]. Optical assays were featured prominently in this review [165]. Nevertheless, as recently as five years ago, they also commented that “as of now, there is no single method that is rapid, non-invasive, handles large sample numbers, and provides accurate measure of cell wall lignin contents” [165]. Infrared and near-IR spectra offer a non-destructive analysis of lignin (similar to Raman) in wood and fibers, including guaiacyl and syringyl content. However, neither near-IR nor Raman techniques satisfy several other deliverables in the above commentary. The near-IR/IR spectral data informs on the types of functional groups such as carbonyls or carboxyls. For example, near-IR spectral analysis and data mining with multivariate data analysis (MVDA) was used for characterizing the physical and chemical properties of hardwoods such as oak and poplar. Measurements included total and acid soluble lignin content and these data had a moderate-to-high correlation with predicted values [166]. Light scattering techniques including MALLS, quasi-elastic light scattering (QELS) and interferometric refractometry were used to monitor changes in lignin conformation over time. The changes included differences in molecular mass, radius of gyration and hydrodynamic radius [167]. It will be recalled that such measurements can also be used to study the fractal properties of lignin.

Unique UV-visible (UV-Vis) signatures of lignin can be a useful tool for lignin analysis. However, determination of the extinction coefficient is problematic since it cannot be assumed that the extinction coefficients determined for the monolignols will be applicable to the polymerized lignin [165]. Lignin was localized in woody tissue using UV microspectrophotometry using 280nm wavelength light [168]. The technique was reported to be semi-quantitative and was based upon the UV illumination of 1 micron thick woody tissue sections for enabling lignin localization.

### 9.3. Scanning Probe Microscopy (SPM)

Scanning probe microscopy or SPM is an umbrella term that captures a variety of microscopy techniques including TEM, SEM, NSOM, AFM, tip-enhanced Raman spectroscopy and coherent anti-Stokes Raman scattering (CARS) microscopy. The applications of SPM as they relate to biomass dissolution were reviewed recently [169]. The AFM technique is a powerful tool for biomolecular imaging including polymeric lignin due to its potential for atomic level resolution and overlaps perfectly with the nanometer scale of plant cell wall components including lignin. The NSOM technique has somewhat coarser resolution in the 100 nm range. The AFM tip is used to measure the surface properties whereby a laser spot is reflected as the tip is rastered across the sample surface providing a topographical map. With NSOM, an optical fiber probe is used for resolution at the hundreds of nanometers scale [169]. 

### 9.4. NMR

Two dimensional NMR has emerged as a powerful technique for the fine resolution structural characterization of wood wall polymers. When MWL isolated using a 4:1(v/v) mixture of dimethylsulfoxide (DMSO) and 1-methylimidazole was used for 2*D* NMR analysis, the data coupled with nanoindentation became representative of *in vivo* lignin. The technique of solution state 2*D* NMR was used for further characterization *via* heteronuclear single quantum coherence (HSQC) and gave excellent peak separation of aromatic and aliphatic correlations of lignins [171]. Both solid and solution state 2-D NMR techniques are well suited for lignin polymer analyses. 

### 9.5. Mechanical Properties

Nanoindentation is a technique for the measurement of mechanical properties at the nanoscale, such as elastic modulus, hardness, and strain rate across one micron length scale. The technique involves piercing the test sample with an indenter. Wimmer *et al.* [170] used nanoindentation to study the mechanical properties of plant cell walls including elastic modulus and hardness (or stiffness).

### 9.6. Computational Tools

Fractal analysis of lignin were discussed above (section 8f) [13,14]. Computational approaches were used as early as 1996 for studying LCCs and demonstrated that lignin most likely existed as a helix [172]. Interactions between lignin and carbohydrate supramolecular aggregates were also studied using molecular mechanics and conformational calculations. It was proposed that both lignin and carbohydrates undergo reciprocal interactions including twisting and coiling around a lignin helix and thereby condensing the complex. These properties influenced the eventual supramolecular organization of LCCs [172] that in turn determined the physical properties of wood such as mechanical strength. Energy minimization analysis of lignin using computational tools also revealed the 3*D* nature of lignin to be helical [173]. Several types of lignin conformers might exist *in planta* some of which could be helical as predicted by computational calculations. Computational and modeling tools are complementary to physicochemical studies for the elucidation of lignin structure and function.

In conclusion, there is no single technique that will clarify all the complexities of lignin structure and conformation. Instead, a suite of physicochemical, computational tools, techniques and an integrated approach are required for analyzing the supramolecular self-assembled complex of lignin, especially *in planta*.

## 10. Lignin Deconstruction—Nature’s Instructions

### 10.1. Lignin Barrier

We now turn to delignification strategies by taking lessons from nature. There are several reasons for the barrier properties of lignin [23,62,106]. These include: 1) Lignin wraps around portions of carbohydrates, becoming a physical barrier to cellulases and β-glucosidases, thereby preventing these enzymes from accessing cellulose and cellobiose. 2) Lignin interacts covalently and non-covalently with carbohydrates blocking the access to carbohydrate degrading enzymes. 3) Lignin, due to its hydrophobic polyphenolic character binds to carbohydrate enzymes resulting in non-productive interactions with these enzymes. 4) Lignin, due to its polyphenolic nature, inactivates or inhibits cellulose digesting enzymes through the formation of enzyme-inhibitor complexes. 5) Pre-treatment of biomass produces phenolic and non-phenolic inhibitors that inactivate the carbohydrate hydrolyzing enzymes. 6) Supramolecular organization is an obstacle for cellulases and β-glucosidases whilst accessing their substrates. 7) Lignin also adsorbs these enzymes, physically removing them from their substrate vicinity, and thereby prevents catalysis. 8) The potency of lignin inhibition is dependent on its content, type of lignin (G or GS), its crosslinked, phenolic and polymeric structure. Several biomass pre-treatments have been developed including treatment with acid, alkali, hot water, steam, lime, ammonia and ionic liquids. All have their advantages and disadvantages [79,174,175]. Pre-treatments for biomass dissolution (prior to saccharification) have several hurdles to overcome including cost, toxicity, environmental impact, effect upon downstream processes, and so on [79,163].

### 10.2. Altering Lignin

The complex, chaotic nature of lignin biosynthesis and post-synthesis assembly into the cell wall makes it hard to design fault-free biomass deconstruction strategies. All processes devised so far have their particular strengths and respective disadvantages. The redundancy of enzymes involved in lignin biosynthesis has unpredictable consequences on plant growth or viability, consequent to artificial genetic manipulations. On the other hand, the malleability of lignin offers opportunities for chemical genetics and recombinant DNA methodologies for producing biofuels-friendly plants as a resource for renewable energy production. Thus, changes to the guaiacyl-syringyl content of lignin (Table 1 and Table 2) can be achieved by blocking coniferyl alcohol production or redirecting sinapyl alcohol biosynthesis (Figure 3) [4,9]. This could decrease lignin-influenced biomass recalcitrance, improve deconstruction and eventually the saccharification efficiency. 

Redirecting G/S ratio of lignin might be achieved through down regulation of COMT activity along with CCoAOMT, F5H and CAD activities (Figure 3). On the other hand, PAL/TAL, C4H, C3H and 4CL are not suitable targets for genetic manipulation to alter lignin composition of plants (enzymes that are downstream from CCR) (Figure 3) [30]. Changes to enzyme activities may be achieved by suppressing the appropriate gene(s) expression [12,23,30,35,36,176,177]. Generally, an increase in G/S ratio leads to improved biomass conversion. There are several caveats to these strategies. For example, and as discussed above (section 4d), plants are able to incorporate a variety of phenolics into their structures that go beyond the traditional role of the three monolignols (Figure 1). The redundancy of enzymes involved in lignin biosynthesis is another factor. The susceptibility of genetically engineered plants to biotic/abiotic stressors [30], “normal” growth and development and in extreme instances, even the survivability of the modified plants can be affected [177]. Plants with reduced levels of lignin might become fragile due to a lack of rigidity in the plant wall and result in stunted growth. How such modified plants interact with the environment is unknown and attention should be paid to the sensitivity of the general populace regarding the introduction of genetically modified plants into the environment solely for their use as lignocellulosic biofuels. 

### 10.3. Our Microbial Teachers

As long as there have been wooden structures there have been creatures that fed on the wood, sometimes creating havoc for humans. Microbes in the hindgut of termites are responsible for the attack on homes and ruminal microbes help cattle to digest the cellulose in the grass. Wood decay in natural habitats such as rainforest soil is brought about by fungi (so-called white rot and brown rot) as well as bacteria. These types of microorganisms have evolved naturally for efficiently breaking down lignin, hemicellulose and cellulose and therefore might provide clues for effective deconstruction programs in lignocellulosic biomass conversion. 

Certain members of *basidomycetes*, the white rot fungi, are amongst the most efficient degraders of lignin found in nature [18]. Despite years of study, lignin degradation by microorganisms is still poorly understood [36]. White rot fungi release a battery of enzymes that act in concert to degrade lignin to CO_2_ and H_2_O. These fungi act most frequently upon angiosperm wood and often leave the white carbohydrates behind (hence the name, white rot fungi) [15]. Brown rot fungi grow on gymnosperm wood and consume the carbohydrates leaving the oxidized brown lignin behind (hence, brown rot fungi). Within the *basidomycetes* white rot fungi, the most efficient specimens include *Phanerochaete chrysosporium* and *Trametes versicolor* [36]. These organisms produce different enzymes including a series of redox enzymes such as peroxidases, laccases and polyphenol oxidases. 

In addition to direct oxidative effects, these enzymes also use RSMs which are small molecules that assist in oxidative functions. It is reasoned that the large, polymeric lignin is unable to fit into the catalytic pocket of an enzyme and therefore the RSMs are an active participant and an important intermediary in the enzymatic breakdown of lignin [5]. A number of small molecules including the monolignols themselves (such as coniferyl alcohol or veratryl alcohol; Figure 1) have been proposed to act as RSMs [28,45,46,47,178,179,180,181,182]. Indeed, such RSMs might be invaluable for developing an inexpensive, nontoxic way of degrading lignin. The genome of *Phanerochaete chrysosporium* was the first to be sequenced amongst white rot fungi [183]. Such molecular biology developments will aid in the design of more potent lignin degrading enzymes. 

A third strategy for improving lignin degradation might be to grow microorganisms in the presence of lignin compounds as the sole carbon and energy source and then to isolate the strains using such materials capably. The enzymes secreted by such organisms are expected to depolymerize and degrade lignin efficiently [184]. Finally, isolates from the rainforest microbial communities and lab-grown microorganisms are a resource for lignin degrading enzymes. An integrated approach is required with an interdisciplinary (molecular genetics, various Omics, informatics, synthetic biology, chemistry, biochemistry, microbiology, and so on) team of dedicated scientists to achieve the goal of sustainable, cost-effective supply of lignocellulosic biofuels. The complexity of the lignocellulosic biofuels problem leaves little room for an individualized or a fragmentary approach. 

### 10.4. Delignifying Enzymes

It is a paradox of lignin biochemistry that the same types of enzymes are suspected to be involved in both its biosynthesis and breakdown, being both the creators and the destroyers of the biopolymer. It appears that context is everything in lignin chemistry and biology. The enzymes involved in lignin degradation include peroxidases, laccases and polyphenol oxidases. It may be recalled that peroxidase and laccase radicalize the monolignols and enable lignin polymerization (Figure 4; also sections 4c and 4d). How the fully formed polymer escapes attack from these enzymes is not well understood; perhaps the formation of a supramolecular architecture with carbohydrates reciprocally protects the lignin. Another unusual feature of lignin biochemistry is that no authentic plant enzymes have yet been described that can depolymerize and/or degrade lignin. Knowledge of lignin degradation comes largely from observations made with wood decaying microorganisms described in section c) above. Lignin depolymerizing enzymes are a vast field of research and there hundreds of original papers and numerous reviews; thus, only a sampling of the citations are provided here [6,15,18,32,179,185,186].

Peroxidases are probably the single largest family of enzymes that are implicated in lignin degradation. A well characterized example of peroxidases is the enzyme isolated from horseradishes. The enzyme requires H_2_O_2_ as a cofactor where the molecule oxidizes the Fe(III) (ferric) form of the peroxidase in a two electron oxidation resulting in the enzyme intermediate confusingly termed as “Compound I.” Compound I accepts an electron and a proton from a substrate such as a monolignol and converts the monolignol into a radical. During this process “Compound I” is converted to “Compound II,” the oxyferryl iron intermediate. The reduction of “Compound II” in an one electron reaction with a second substrate molecule restores the Fe(III) form of the enzyme, thereby completing the cycle and generating a new radical in the process [6]. Nevertheless, horseradish peroxidases (HRP) appear to be incapable of oxidizing the lignin polymer [5] although most DHP studies have employed HRP to oxidize coniferyl alcohol. The HRP displays poor oxidative capacity towards sinapyl alcohol [187]. Sasaki *et al.* [187] have described a cell wall-bound peroxidase that oxidized sinapyl alcohol and polymeric lignin. 

Lignin peroxidases (LiP; EC 1.11.1.14; previously called “ligninases”) secreted by white rot fungi are able to oxidatively cleave carbon-carbon and ether bonds (Figure 5). The enzyme, similar to HRP, catalyzes the H_2_O_2_-dependent oxidation of lignin [15]. Additionally, the enzyme can oxidize a variety of different phenolic compounds. Another type of peroxidase is Manganese Peroxidase (MnP) (EC 1.11.1.13). This enzyme does not directly oxidize lignin, but instead oxidizes manganese (Mn^2+^ is oxidized to Mn^3+^) and the Mn(III) oxidizes a variety of phenolic substrates and lignin model compounds [15]. Thus, Mn(III) is a RSM. A different flavor of peroxidase is the Versatile Peroxidase (VP) (EC 1.11.1.16) which is a hybrid of LiP and MnP. The VP acts on manganese but also oxidizes a variety of phenolics and non-phenolics independently along with the oxidation of substituted phenols. The last enzyme to be discussed here is Laccase (EC 1.10.3.2), a copper containing enzyme that performs one electron oxidization of phenolic and non-phenolic substrates, while reducing O_2_ to H_2_O [188]. Laccase also has demethylating and demethoxylating activities [188]. Lignin degrading fungi produce laccase under ligninolytic conditions hinting at the possibility that laccases play a role in lignin degradation [188]. In addition to these enzymes, several others have been implicated in breaking down lignin all of which are redox enzymes and some of which generate H_2_O_2_ and the peroxide molecule reduces lignin, further promoting polymer degradation. Some of the oxidases that are suspected to play a role in lignin degradation include glyoxal oxidase, veratryl oxidase and aryl alcohol oxidase [15]. Identifying efficient lignin degrading enzymes is clearly work in progress.

## 11. Conclusions

We have described the properties of lignin, its association with cell wall polysaccharides and its role in biomass recalcitrance influencing cost-effective lignocellulosic biofuels production. We hope that this review will benefit the researcher interested in lignin, biofuels or self-assembly. Conventional crops such as corn or sugarcane have not been the biofuels panacea as originally thought. They also had unexpected consequences on food consumption/price, leading to food *versus* fuel debate, deforestation, land and water use globally. A viable biofuels program should be based on renewable energy resources, be cost-effective, reduce carbon footprint and in the near-term complement existing fossil fuel applications, such as transportation. Theoretical projections are 130 billion gallons of fuel grade ethanol from 1.3 billion tons of biomass (100 gallons/ton) that the United States produces annually [189]. However as of this writing, theory seems unlikely to transform into reality in the near future (5–10 years). A cost-effective lignocellulosic biofuels program must first solve the formidable challenges that are technical, logistical, financial, political and infrastructural in nature [190]. Eliminating the lignin barrier is simply an important first step in this direction.
